# Comparing teacher-made and AI-assisted teaching videos for thyroid eye disease: an exploratory study

**DOI:** 10.3389/fcell.2026.1963967

**Published:** 2026-10-21

**Authors:** Shu Liu, Yue Wang, Yuqing Chen, Yunyun Chang, Ying Wang, Yu Gao, Ruili Wei

**Affiliations:** 1 Department of Ophthalmology, Changzheng Hospital of Naval Medical University, Shanghai, China; 2 Department of General Practice, Changzheng Hospital of Naval Medical University, Shanghai, China

**Keywords:** AI-assisted video, generative AI, medical education, ophthalmology residency training, thyroid eye disease

## Abstract

**Background:**

Thyroid eye disease (TED), together with its acute and vision-threatening complication, dysthyroid optic neuropathy (DON), requires timely diagnosis and intervention. The limited availability of specialists in orbital diseases poses a significant challenge to ophthalmology residency training. In this context, generative artificial intelligence (AI) has created new opportunities for medical education, though its effectiveness in ophthalmology training remains to be explored.

**Objective:**

This exploratory study aims to evaluate the effectiveness of generative artificial intelligence (AI) in ophthalmology education, focusing on TED domains.

**Methods:**

The 46 residents (28 general residents and 18 ophthalmology residents) were randomly assigned to either an AI-assisted or a teacher-made 5-min teaching video on TED. All participants then completed three tests through online questionnaire experiments, each consisting of 20-item clinical questions about TED. Test 1 served as a baseline assessment of disease knowledge, Test 2 was conducted 5 min after learning to evaluate short-term learning outcomes, along with a questionnaire survey about AI tools, and Test 3 was administered 1 week later to assess long-term learning outcome.

**Results:**

Viewing the AI-assisted video significantly improved test scores from baseline to 5 min (mean difference = 13.04; p = 0.004), but the improvement was not sustained at 1 week (mean difference = 7.61; p = 0.16). Completion time also decreased significantly from baseline to both 5 min (p = 0.009; GMR = 0.57) and 1 week (p < 0.001; GMR = 0.45). No significant differences were observed between the AI-assisted and teacher-made video groups across all assessments (all p > 0.05). Additionally, learning experience was positively correlated with learning outcome (r = 0.60; P < 0.001).

**Conclusion:**

Our findings suggested that the AI-assisted and teacher-made videos for TED education showed no statistically significant difference. However, substantial expert oversight was required, and the AI-assisted workflow did not reduce total production time in this study. Given the limited long-term retention, such AI-assisted videos may be better positioned as supplementary resources combined with human expertise.

## 1 Introduction

Thyroid eye disease (TED), also known as thyroid-associated ophthalmopathy (TAO), is a progressive autoimmune disorder characterized by orbital inflammation. It can lead to eyelid retraction, edema, proptosis, restricted ocular motility and diplopia, thereby significantly impacting quality of life (Taylor et al., 2019). However, despite the relatively high prevalence of TED (Bartalena et al., 2021), its management remains challenging due to the diverse symptoms (Smith, 2022), often leading to delays in diagnosis (Khabaz et al., 2026). A United Kingdom study revealed that over half of patients with TED were initially misdiagnosed, and 45% of patients had experienced symptoms for more than 6 months before receiving a diagnosis (Estcourt et al., 2009). Furthermore, the shortage of TED specialists has delayed timely treatment in some cases, ultimately resulting in irreversible vision loss (Burch et al., 2022; Uribe-Leitz et al., 2018). Therefore, it is crucial to provide specialized training for residents to ensure accurate diagnosis and effective treatment of TED.

Currently, the physician-to-patient ratio in China is 1/950, which is well below international standards (Netease, 2023). Due to their heavy clinical duties, senior medical professionals are often unable to provide sufficient guidance to residents, which poses a significant obstacle to the delivery of high-quality medical education. In recent years, artificial intelligence (AI) tools and social media platforms such as YouTube and TikTok have demonstrated significant potential to enhance medical education (Nguyen and Diederich, 2023; Orús et al., 2016). The 2016 Horizon Report recognized AI as a significant advancement, forecasting its substantial influence on higher education (Johnson et al., 2016). Furthermore, the efficacy of video-based online instruction has been well established by academic institutions as a robust supplementary resource for teachers and educational specialists (Ruip´erez-Valiente et al., 2022). Recently, an increasing number of pilot initiatives have demonstrated the value of generative AI across the full spectrum of physician training (Jeon et al., 2023; Lim et al., 2023; Netland et al., 2025), from undergraduate medical education, graduate education, and post-residency specialty fellowships stages (Yuan et al., 2025). In particular, several ophthalmology-specific studies have validated the capacity of these AI tools to master standardized medical assessments (Wu et al., 2025; Wei et al., 2025; Cai et al., 2023; Fiedler et al., 2024), to perform clinical analysis (Xu et al., 2020; Cao et al., 2025; Kang et al., 2025; Shao et al., 2025), and to deliver personalized learning mentorship (Sevgi et al., 2024; Wu et al., 2024; Divito et al., 2023) in ophthalmology.

Although AI has been increasingly applied to the clinical management of TED (Yi et al., 2024), its role in medical education for TED has not been systematically evaluated. TED is characterized by both chronic progression and acute episodes of dysthyroid optic neuropathy (DON), making it representative for this investigation. For the residents, generative AI was used to assist in developing a learning outline and audiovisual materials based on identified knowledge gaps, followed by expert review and curation. This exploratory study aimed to assess the educational feasibility of an AI-assisted, expert-curated teaching video for ophthalmology training and investigate its potential role as a supplementary educational resource.

## 2 Materials and methods

### 2.1 Sample size estimation

The sample size was calculated for the primary endpoint, which was the within-group change in test scores from baseline to 5 min in the AI-assisted video group. Assuming an 8-point improvement (approximately 10% of the total score), a standard deviation (SD) of 8, a two-sided α of 0.05, and 80% power, 16 participants per group were required for a paired t-test. Accounting for a 20% dropout rate, at least 20 participants were required per group. The between-group comparison (AI-assisted vs. Teacher-made video) was a secondary, exploratory endpoint.

### 2.2 Participant recruitment and study design

In this study, we recruited 32 general residents and 20 ophthalmology residents at Shanghai Changzheng Hospital in November 2025. Eligible residents were those who provided written informed consent and had less than 5 h of prior systematic training in TED. Exclusion criteria were failure to complete all three test rounds or a test completion time of less than 120 s. The <120-s threshold was established prior to data analysis but was not pre-registered; it was based on the consideration that the 20-item test required a minimum of approximately 2 min for meaningful completion. All participants completed three rounds of online tests through the survey platform “Wenjuanxing” (Guo et al., 2021; www.wjx.cn) via the social media platform WeChat from November 2025 to January 2026. Each of the three tests consisted of 20 questions covering key domains of TED (anatomy, clinical manifestations, diagnosis, and treatment). Test 1 was conducted in early December 2025 to collect baseline demographic information (e.g., age, gender, and department) and to assess the baseline knowledge lof TED (Supplementary Table S1).

Based on the results of Test 1, two teaching videos were produced: one by generative AI and the other by ophthalmology teachers. And then two online tests were conducted using different 20-item single-choice questionnaires at 5 min after learning (Test 2, in late December 2025) and 1 week after learning (Test 3, in January 2026), with structures parallel to those of Test 1. The 5-min post-test was considered an immediate/short-term learning outcome, and the 1-week test was considered a delayed/long-term retention outcome, consistent with prior memory research defining 1 week as the shortest interval to capture long-term retention (Galusca et al., 2026; Peng et al., 2024). To ensure comparable difficulty levels across the three tests, each test was designed to include 35% easy, 35% moderate, and 30% difficult questions, based on the correct-response rates from the Chinese Ophthalmology Residency Training Examination. A panel of six TED experts evaluated the content validity of the three tests using a 5-point Likert scale (1 = strongly disagree, 5 = strongly agree). The item-level content validity index (I-CVI) was calculated as the proportion of experts rating an item as 4 or 5. The scale-level content validity index (S-CVI/Ave) was then computed as the average of all I-CVI values. Content validity was considered acceptable when S-CVI ≥ 0.90 and I-CVI ≥ 0.78 (Polit et al., 2007). In addition, item-level difficulty (p) and discrimination were calculated for each of the 20 items in each test form. Difficulty was defined as the proportion of correct responses. Discrimination was assessed using the upper–lower 27% group method and point-biserial correlation.

Participants’ demographic information, test scores, completion times and feedback on the medical instructional videos were collected after the final online experiment and stored in Microsoft Excel 2019 for subsequent analysis.

### 2.3 Production of teaching videos

In this experiment, videos were produced with and without AI assistance and labeled as “AI-assisted” and “teacher-made”. Research has shown that DeepSeek-V3, a cost-effective Chinese large language model (LLM) (Gibney, 2025), performs as well as, and in some cases better than, proprietary LLMs in clinical decision-making (Sandmann et al., 2025). Data collected from Test 1 were shared with both DeepSeek and two experienced orbital disease specialists to identify gaps in participants’ knowledge. For the AI-assisted video, DeepSeek was subsequently prompted with the following request: “Can you create a script for an educational video about TED based on the results of the aforementioned questionnaire?” DeepSeek then generated corresponding subtitles and text content for visual illustrations in real time, which were subsequently used as input for the Dreamina AI platform (dreamina.capcut.com, Seedream 3.0) to generate visuals, including pictures and video segments (Supplementary Table S2). For DeepSeek-V3 and Dreamina, default platform settings were used (no user-configurable parameters were available). The figures and video segments in this study were created using Dreamina AI (https://dreamina.capcut.com). According to the platform’s terms of service, users retain the intellectual property rights to both their input content and the corresponding AI-assisted outputs, within legal limits.

Several of the generated images are shown in Figure 1. The generated visual content often exhibited notable variability and occasional inappropriateness, necessitating more careful screening and guidance than the text content generated by DeepSeek. The expert review process is described in the step 2 of Supplementary Table S2. The selected Dreamina AI-assisted visuals and AI-assisted voiceovers were then incorporated into a teaching video (URL: https://youtu.be/uXtQkTqtRuI) using CapCut tool (www.capcut.com, version 7.9). It took the specialists 2.5 additional hours reviewing and correcting the content-specifically, correcting inaccuracies, replacing distorted images, adjusting voiceover pacing and adding annotations. The total human effort for the AI video was approximately 6.5 h.

**FIGURE 1:**
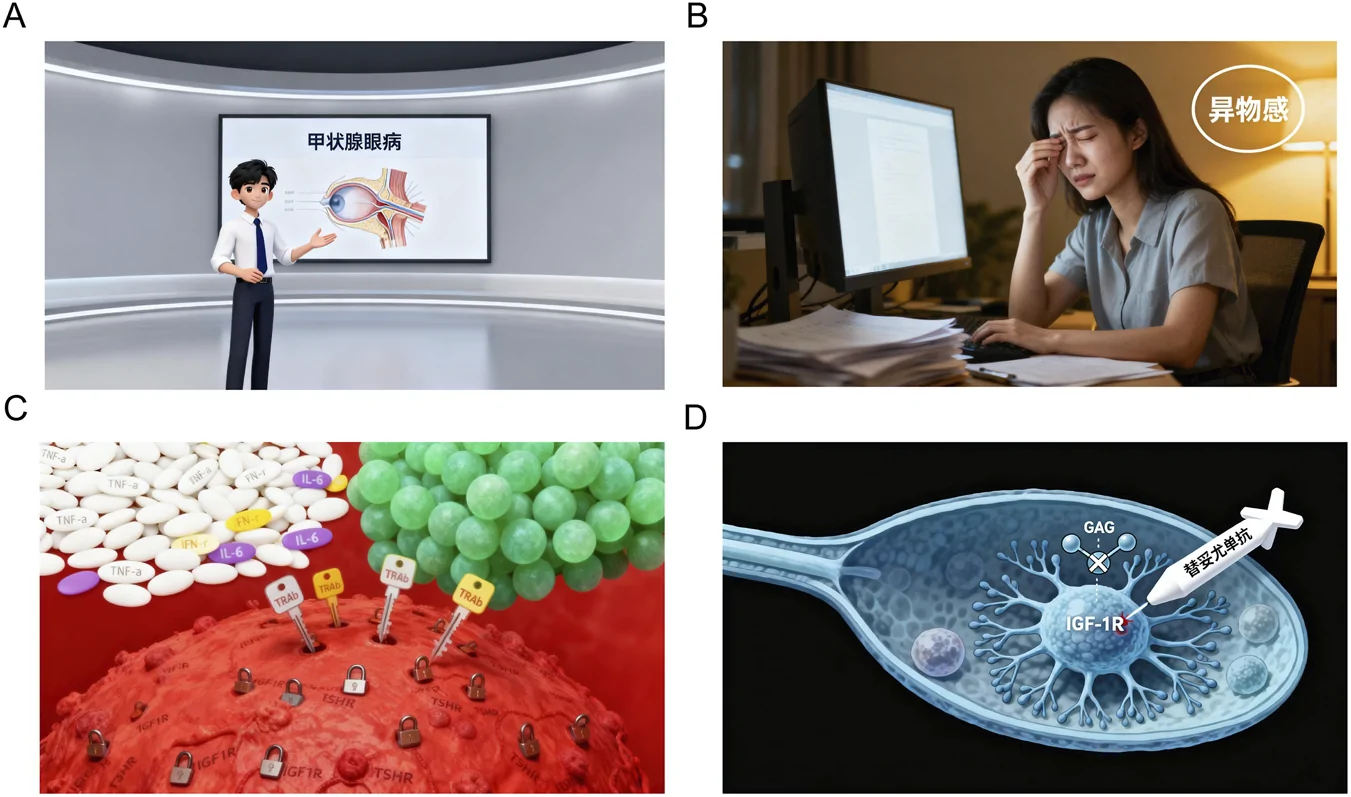
Several images in AI-assisted teaching video. **(A)** A virtual lecturer explains TED in a studio. **(B)** A female TED patient with a pained expression in the early to middle stages. **(C)** TRAb triggers cytokine release by binding to IGF1R/TSHR. **(D)** The mechanism of action of teprotumumab.

The production of the teacher-made video involves three steps: developing a structured outline based on the results of Test 1, designing a PowerPoint presentation, and incorporating the teacher-created subtitles and voiceover into the educational video using CapCut (URL: https://youtu.be/wBXLys3N9is). The teacher was provided with standard office hardware and was given 4.5 h to complete the video. Both videos covered the main topics of TED, including anatomy, pathogenesis, clinical manifestations, differential diagnosis, and treatment. The duration was 5 min 8 s for the AI-assisted video and 5 min 12 s for the teacher-made video. Both videos were finally uploaded to an unlisted page on YouTube (Netland et al., 2025), and the corresponding links are provided as provided above.

### 2.4 Content validity of teaching videos

The content validity of the video assessment scale was evaluated using a panel of 6 experts (4 senior ophthalmologists specializing in TED and 2 medical education specialists). Each expert rated 12 items across 3 dimensions, including clinical accuracy, visual fidelity and pedagogical appropriateness by a 5-point Likert scale (1 = strongly disagree, 5 = strongly agree).

### 2.5 Quality evaluation of teaching videos in online experiments

Following stratification by residency type (ophthalmology vs. general), participants within each stratum were randomly assigned in a 1:1 ratio to either the AI-assisted video group or the teacher-made video group using a random number table generated by R (version 4.5.2). To ensure allocation concealment, the randomization sequence was generated by an independent researcher who was not involved in participant recruitment or outcome assessment. Group assignments were placed in sequentially numbered, opaque and sealed envelopes. After participants completed the baseline assessment (Test 1), the corresponding envelopes were opened by a study coordinator to reveal the group assignment.

Accordingly, residents watched either the teacher-made or AI-assisted educational video. The testing flowchart and the learning process are detailed in Figure 2. Due to the nature of the intervention (viewing different types of videos), it was not possible to blind the participants or the interventionists. However, the outcome assessors (data analysts) were blinded to the group assignments throughout the analysis phase.

**FIGURE 2:**
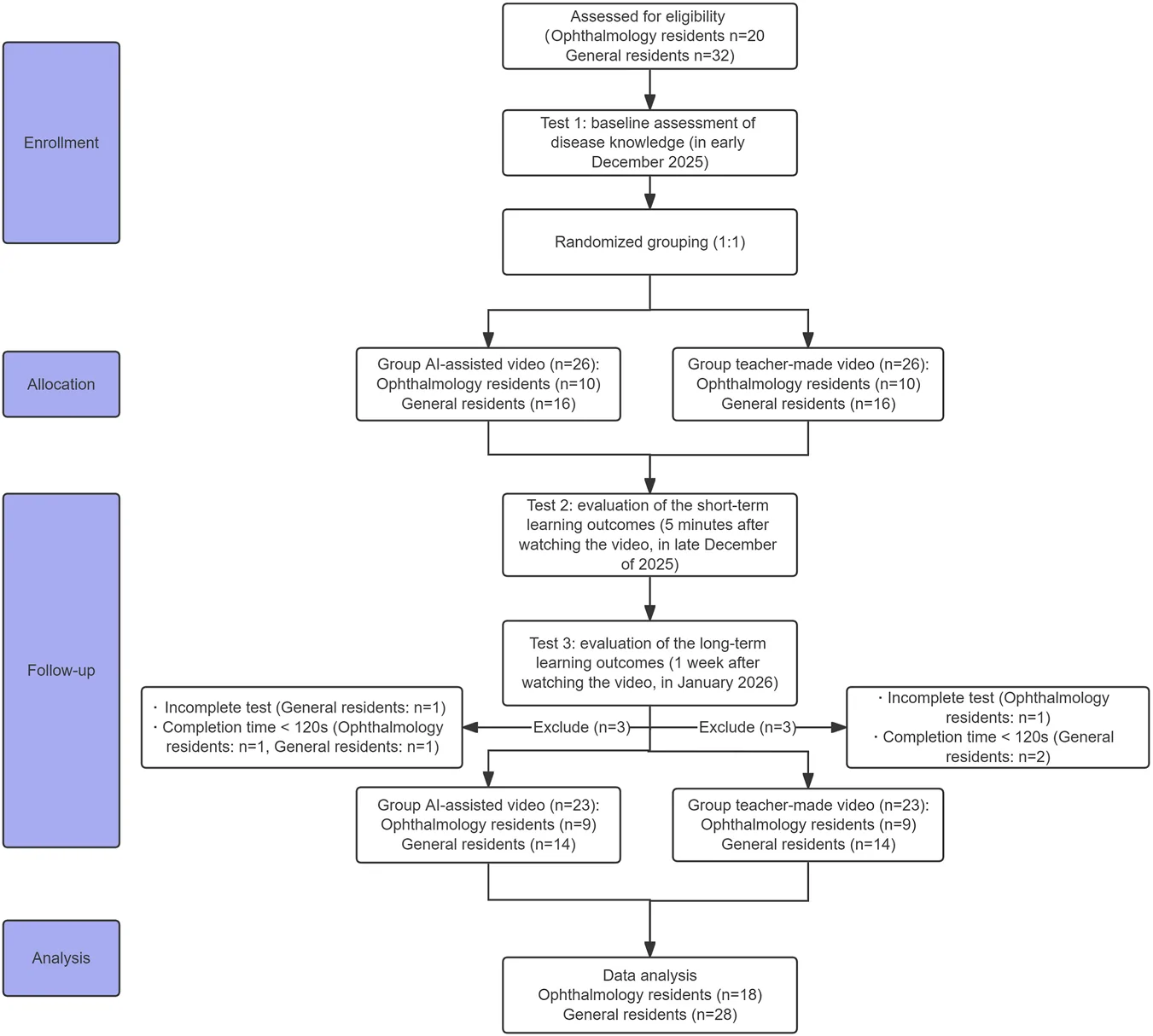
The chronological flowchart for the three tests and the learning process in the two groups.

Test 2 was conducted 5 min after the video, in late December 2025, and included questions on learning experience and attitudes toward AI (Supplementary Table S3). Test 3 was completed 1 week later in January 2026 (Supplementary Table S4). Instructional impact was assessed via score improvements and time reductions. Additionally, participants’ perceptions of AI-assisted teaching were collected using a 13-item scale assessing four dimensions: topic familiarity, learning outcome, learning experience, and optimism about AI, along with an optional open-ended question on video improvement: “What is the aspect of the instructional video most in need of improvement?” Two independent coders (research assistants with backgrounds in educational psychology) coded the responses to the open-ended question. Discrepancies were resolved through consensus discussion.

To prevent disclosure of correct responses from influencing subsequent assessments, correct answers and explanatory materials were hidden from participants during all assessments and were released only after completion of all study assessments.

### 2.6 Statistical analysis

Continuous variables were summarized as mean ± standard deviation (SD) or median with interquartile range (IQR), as appropriate. For test scores, linear mixed-effects models (LMMs) were fitted with group, time (baseline, 5 min, 1 week), group × time interaction, and residency type as fixed effects, and subject-specific random intercepts. Between-group comparisons at each time point were based on model-estimated marginal means and were Bonferroni-adjusted across the three time points; within-group comparisons were derived from the same LMMs and Bonferroni-adjusted within each group across the three time-points. For test scores, effect sizes were reported as Cohen’s d with 95% confidence intervals (CIs).

For completion time, which was positively skewed, a natural log transformation was applied. Between-group comparisons were performed using LMMs that included log-transformed baseline completion time as a covariate and only follow-up time points (5 min and 1 week) to avoid collinearity, thereby adjusting for baseline differences between groups; results were back-transformed and reported as geometric mean ratios (GMRs) with 95% CIs, with Bonferroni adjustment across the two follow-up time points. Within-group changes in completion time were assessed using a change score model, with the difference between log-transformed follow-up and baseline completion time as the dependent variable (log change = log [follow-up] minus log [baseline]). The model included group, time (5 min and 1 week), group × time interaction, and residency type as fixed effects, plus subject-specific random intercepts. The log change was tested against zero for each group and time point, with Bonferroni adjustment within each group across the three pairwise comparisons (5 min minus baseline, 1 week minus baseline, 1 week minus 5 min). The group × time interaction in this model was used to assess whether the magnitude of change from baseline differed between groups. Within-group results were back-transformed and reported as GMRs (follow-up-to-baseline ratios) with 95% CIs.

All LMMs used maximum likelihood estimation and included all available data under the missing-at-random assumption; no imputation was performed for the primary analyses. Subgroup analyses by residency type were exploratory and hypothesis-generating only; no formal interaction tests were performed because the study was not powered for such analyses. All tests were two-sided. Statistical significance was defined as a Bonferroni-adjusted p < 0.05.

All statistical analyses were conducted in R 4.5.2. Graphical presentations were generated using GraphPad Prism 9.5.0 for Windows.

### 2.7 Ethical approval

Ethics approval for this study was obtained from the Biomedical Research Ethics Committee of Changzheng Hospital (Identifier: 2021SL044). Each participant signed an informed consent form detailing the purpose, procedures, risks, and their right to withdraw without penalty. A copy of the consent form is provided in Supplementary Table S5. All data were deidentified to protect participants’ privacy and confidentiality.

## 3 Results

### 3.1 Participants’ demographic characteristics

A total of 46 participants (28 general residents and 18 ophthalmology residents) were included in the final analysis. We excluded 1 general resident and 1 ophthalmology resident who did not complete all three rounds of testing, as well as 3 general residents and 1 ophthalmology resident whose testing times were less than 120 s. As shown in Table 1, both the AI-assisted and teacher-made groups comprised 9 ophthalmology residents and 14 general residents. The mean age of the participants was 27.00 ± 3.86 years, with no significant difference in mean age between groups (AI-assisted group: 27.13 ± 4.14 years vs. teacher-made group: 26.87 ± 3.65 years, p = 0.77, Table 1). There was no statistically significant difference in gender between the two groups (p = 0.56).

**TABLE 1:** Personal information on residents participating in the online questionnaire experiment.

Variable	All (n = 46)	AI-assisted (n = 23)	Teacher-made (n = 23)	p value
Age (years)	27.00 ± 3.86	27.13 ± 4.14	26.87 ± 3.65	0.77^a^
Gender (M/F)	24/22	11/12	13/10	0.56^b^
Residency type (ophthalmology/General	18/28	9/14	9/14	>0.99^b^

Continuous covariates are shown as mean ± SD and categorical covariates are shown as number of events observed. p values.

^a^ a Mann-Whitney U test.

^b^ Chi-square test.

### 3.2 Content validity of teaching videos

Before incorporating the teaching videos into the study, we assessed their content validity. The results showed that 11 of the 12 evaluation items met the prespecified item-level CVI criterion for the AI-assisted video. One visual-fidelity item, concerning the accuracy of pathological changes and freedom from distortion, had an I-CVI of 0.67 (Supplementary Table S6). All 12 items of the teacher-made video achieved an I-CVI≥0.78 (range: 0.83–1.00). The overall S-CVI/Ave was 0.93 for the AI-assisted video and 0.97 for the teacher-made video, both exceeding the recommended threshold of 0.90. This confirmed that the scale items appropriately captured the core dimensions of video quality. Based on this validated tool, experts rated both videos highly, with mean scores of 4.43/5 for the AI-assisted video and 4.56/5 for the teacher-made video. Item-level mean scores ranged from 3.83 to 5.00 for the AI-assisted video and 4.17 to 5.00 for the teacher-made video.

### 3.3 Validity of the three tests

The content validity of the three tests was assessed using I-CVI and S-CVI/Ave, as shown in Supplementary Table S7. The S-CVI/Ave was 0.97 for Test 1, 1.00 for Test 2, and 0.97 for Test 3, with all items meeting the I-CVI ≥0.78 threshold. This indicates excellent inter-rater agreement and robust content validity for all three tests. Experts rated the three tests as high in quality and comparable in difficulty, with item-level mean scores ranging from 4.17 to 4.83 for Test 1, 4.33 to 4.67 for Test 2, and 4.17 to 4.67 for Test 3.

The internal consistency of the three tests was assessed using the Kuder—Richardson Formula 20 (KR-20), which a special case of Cronbach’s α for dichotomously scored items. The KR-20 coefficients were 0.80 for Test 1, 0.76 for Test 2, and 0.82 for Test 3, all exceeding the commonly accepted threshold of 0.70, indicating acceptable to good internal consistency across the three tests.

Construct validity was examined using exploratory factor analysis (EFA). The Kaiser—Meyer—Olkin (KMO) measures were 0.544 for Test 1, 0.617 for Test 2, and 0.613 for Test 3, with Test 1 falling below the recommended threshold of 0.60. Bartlett’s test of sphericity was significant for all three tests (Test 1: χ^2^ = 327.865, df = 150, p < 0.001; Test 2: χ^2^ = 351.570, df = 171, p < 0.001; Test 3: χ^2^ = 287.239, df = 105, p < 0.001), providing supportive evidence of inter-item correlations. The modest KMO values, particularly for Test 1, likely reflect the limited sample size (n = 46) and the multidimensional nature of the knowledge domain, which covers multiple areas such as pathogenesis, diagnosis, and treatment.

As shown in Supplementary Table S8, mean item difficulty was 0.67, 0.79, and 0.70 for Tests 1, 2, and 3, respectively. Mean discrimination indices (upper–lower 27%) were 0.48, 0.40, and 0.52, and mean point-biserial correlations were 0.35, 0.34, and 0.41. These results indicate acceptable and broadly comparable psychometric properties across the three forms. Item-level parameters are also presented in Supplementary Table S8. A few items showed low or negative point-biserial correlations, which may reflect small sample size or item ambiguity.

### 3.4 Effect of AI-assisted and teacher-made teaching video

To assess the effects of the teaching videos, we analyzed the changes in test scores across three consecutive testing rounds. Primary analyses were based on 46 participants (23 per group). Bonferroni correction for multiple comparisons was applied. As shown in Figure 3A, in the AI-assisted group, test scores increased significantly from baseline to 5 min (Test 2 minus Test 1: mean difference = 13.04, 95% CI: 3.51 to 22.57; p = 0.004; Cohen’s d_rm = 0.61), but the change from baseline to 1 week was not statistically significant (Test 3 minus Test1, mean difference = 7.61, 95% CI: −1.92 to 22.57; p = 0.16). In the teacher-made group, a similar pattern was observed: scores changed significantly from baseline to 5 min (Test 2 minus Test1: mean difference = 11.52, 95% CI: 1.99 to 21.05; p = 0.01; Cohen’s d_rm = 0.76), whereas the change from baseline to 1 week was not significant (Test 3 minus Test1: mean difference = 4.78, 95% CI: −4.75 to 14.31; p = 0.67). The group × time interaction was not statistically significant (p > 0.05), indicating similar trajectories between groups.

**FIGURE 3:**
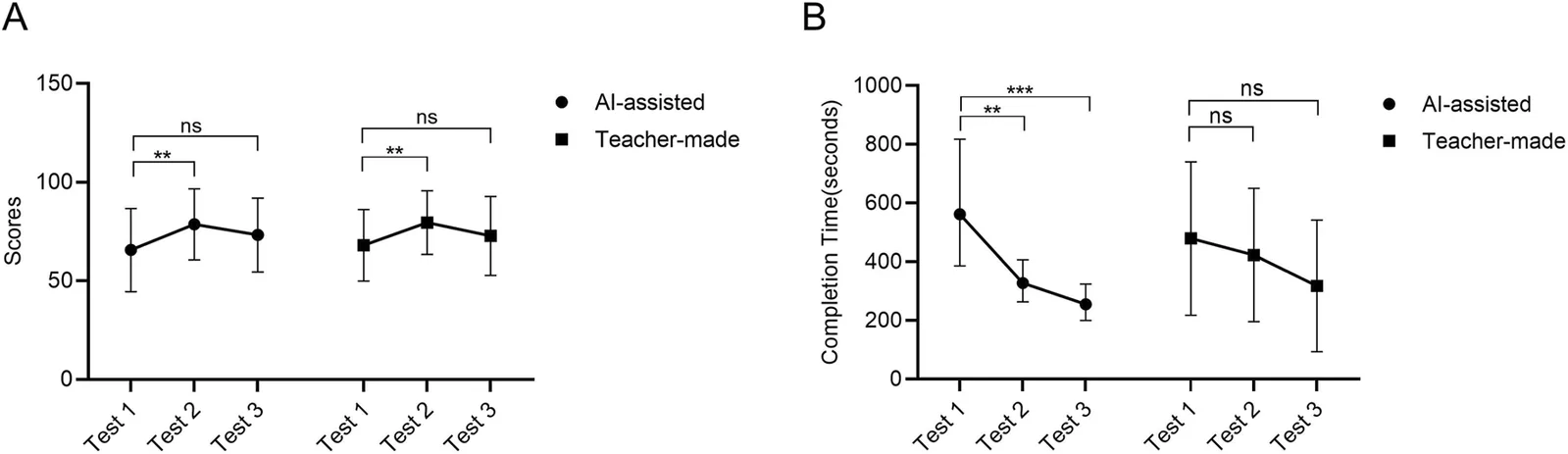
Changes in test scores and completion time across three tests. **(A)** Test scores are presented as means; error bars represent SD. **(B)** Completion time is presented as geometric means; error bars represent 95% CI. In both panels, asterisks indicate Bonferroni-adjusted comparisons versus Test 1 (baseline), derived from linear mixed-effects models: *p < 0.05; **p < 0.01; ***p < 0.001; ns, not significant.

We further analyzed changes in completion time across the three tests. As shown in Figure 3B, in the AI-assisted group, completion time decreased significantly from baseline to 5 min (Test 2 minus Test 1: log difference = −0.57, 95% CI: −0.94 to −0.20; p = 0.009; GMR = 0.57, 95% CI: 0.39–0.82) and from baseline to 1 week (Test 3 minus Test 1: log difference = −0.79, 95% CI: −1.16 to −0.42; p < 0.001; GMR = 0.45, 95% CI: 0.32–0.66). The change from 5 min to 1 week was not significant (Test 3 minus Test 2: p = 0.28). In the teacher-made group, no significant within-group changes were observed (all p > 0.05).

The analysis of between-group differences in test scores (Table 2) revealed no significant differences at baseline (Test 1: mean difference = −4.64, 95% CI: −15.45 to 6.17; p = 0.40), 5 min (Test 2: mean difference = −3.12, 95% CI: −13.92 to 7.69; p = 0.57), or 1 week (Test 3: mean difference = −1.81, 95% CI: −12.62 to 9.00; p = 0.74). The group × time interaction was not statistically significant.

**TABLE 2:** Between-group differences in test scores at each time point (AI-assisted minus teacher-made).

Time point	AI-assisted (n = 23, mean ± SD)	Teacher-made (n = 23, mean ± SD)	Mean difference (AI-assisted minus teacher-made, 95% Cl)	p value (Bonferroni-adjusted)	Cohen’s d (95% Cl)
Test 1	65.65 ± 21.07	68.04 ± 18.14	−4.64 (−15.45 to 6.17)	0.40	−0.35 (−1.17 to 0.47)
Test 2	78.70 ± 18.04	79.57 ± 16.09	−3.12 (−13.92 to 7.69)	0.57	−0.24 (−1.05 to 0.58)
Test 3	73.26 ± 18.68	72.83 ± 20.05	−1.81 (−12.62 to 9.00)	0.74	−0.14 (−0.95 to 0.68)

Mean differences are AI-assisted group minus Teacher-made group; negative values indicate lower scores in the AI-assisted video group. Test 1 = baseline, Test 2 = 5 min, Test 3 = 1 week. p-values are Bonferroni-adjusted across the three time-point comparisons. Cohen’s d is the mean difference divided by the residual SD from the mixed-effects model. SD, standard deviation.

Similar patterns were observed for between-group differences in completion time (Table 3). The median (IQR) completion time at baseline was 522 (284–967) seconds in the AI-assisted group and 471 (330–586) seconds in the teacher-made group. At 5 min, the respective medians were 347 (233–474) and 338 (266–418) seconds, with no significant between-group difference (log mean difference = −0.06, 95% CI: −0.36 to 0.24; p = 0.70; GMR = 0.94, 95% CI: 0.70–1.28). At 1 week, the medians were 253 (195–368) and 241 (181–402) seconds, again without a significant difference (log mean difference = −0.09, 95% CI: −0.39 to 0.21; p = 0.56; GMR = 0.92, 95% CI: 0.68–1.24). The group × time interaction for completion time was not statistically significant.

**TABLE 3:** Between-group differences in completion time at each time point (AI-assisted minus teacher-made).

Time point	AI-assisted (n = 23, median (IQR))	Teacher-made (n = 23, median (IQR))	Log mean difference (AI-assisted minus teacher-made, 95% Cl)	p value (Bonferroni-adjusted)	GMR (95% Cl)	Cohen’s d_log (AI-assisted minus teacher-made, 95% Cl)
Test 1	522 (284–967)	471 (330–586)	—	—	—	—
Test 2	347 (233–474)	338 (266–418)	−0.06 (−0.36 to 0.24)	0.70	0.94 (0.70–1.28)	−0.14 (−0.84 to 0.56)
Test 3	253 (195–368)	241 (181–402)	−0.09 (−0.39 to 0.21)	0.56	0.92 (0.68–1.24)	−0.21 (−0.91 to 0.50)

Data are median (IQR) in seconds. Test 1 = baseline, Test 2 = 5 min, Test 3 = 1 week. Completion time was log-transformed; models included only follow-up time points (5 min and 1 week), with log-transformed baseline completion time as a covariate. Comparisons are AI, minus Teacher. Negative log mean differences (GMR < 1) indicate lower log completion time in the AI-assisted group. p-values are Bonferroni-adjusted. GMR is the primary effect size; Cohen’s d_log is the standardized mean difference on the log scale. GMR: geometric mean ratios.

To address potential attrition bias, we performed an intention-to-treat (ITT) analysis including all 52 randomized participants and an additional sensitivity analysis excluding only participants with incomplete tests (n = 50). Both the ITT (n = 52) and sensitivity (n = 50) analyses yielded results consistent with the primary available-case analysis (n = 46), suggesting that the post-randomization exclusions did not materially alter the conclusions (all p > 0.05 for between-group comparisons; detailed results available in Supplementary Tables S9–S12).

We further evaluated the impact of the video on learning outcomes for DON, as shown in Supplementary Figure S1. However, no statistically significant differences were observed in DON-related accuracy across the three testing rounds, either in the overall cohort (p > 0.05; Cohen’s d ranged from 0.10 to 1.46). Notably, among residents who learned the AI-assisted video, the accuracy of the ophthalmology group on Test 3 (55.60 ± 0.00) was even lower than that on Test 1 (85.20 ± 16.96; p = 0.03). These findings suggest that, overall, neither video format had a significant impact on DON-related learning outcomes.

### 3.5 The comparison of teaching outcomes among subgroups

In exploratory subgroup analyses, as shown in Supplementary Table S13, at baseline, ophthalmology residents in both groups had numerically higher scores than general residents (AI-assisted: 73.33 ± 18.03 vs. 60.71 ± 22.00; teacher-made: 75.00 ± 12.50 vs. 63.57 ± 20.14). In the AI-assisted group, ophthalmology residents showed a sustained numerical improvement at 1 week (mean change 12.78 ± 18.73), whereas general residents showed a smaller and more variable change (mean change 4.29 ± 26.66). In the teacher-made group, ophthalmology residents also showed a numerical increase at 1 week (mean change 13.33 ± 13.23), while general residents showed a slight decrease (mean change −0.71 ± 21.83). However, these subgroup differences were not formally tested, and the small subgroup sample sizes warrant cautious interpretation.

As shown in Supplementary Table S14, at 5 min and 1 week, both ophthalmology and general residents in the AI-assisted group showed negative median changes, meaning shorter completion time. At 1 week, general residents showed a larger reduction than ophthalmology residents: −343.0 s (IQR -801.0 to −86.0) vs. −318.0 s (IQR -691.5 to 7.5). In the teacher-made group, ophthalmology residents showed minimal changes: −61.0 s (IQR -148.0–150.0) at 5 min and −37.0 s (IQR -163.0 to 55.0) at 1 week, whereas general residents showed larger reductions: −125.0 s (IQR -303.0 to −33.2) at 5 min and −209.5 s (IQR -354.0 to −138.0) at 1 week. These observations suggest possible differential patterns by residency type.

### 3.6 Residents satisfaction and evaluation

All participants completed the questionnaire, which assessed four dimensions: topic familiarity, learning outcome, learning experience and optimism about AI (summarized in Figure 4). The reliability coefficients for the overall scale (Cronbach’s α = 0.88) and each subscale were within acceptable ranges (α > 0.6, Table 4). Additionally, construct validity was supported by the KMO measure (0.80) and Bartlett’s test of sphericity (χ^2^ = 292.686, df = 136, p < 0.01). Almost all items across the 3 subscales of learning outcome, learning experience, and optimism about AI had mean scores above 4. The exception was Question 2 of optimism about AI, “I believe the content provided by AI tools is completely accurate.” which received an average score of 3.76. These results indicated that participants gave high marks to the AI-assisted video and considered AI-assisted content effective for learning. Although they generally held a positive attitude toward AI-assisted learning, they remained critical of its accuracy.

**FIGURE 4:**
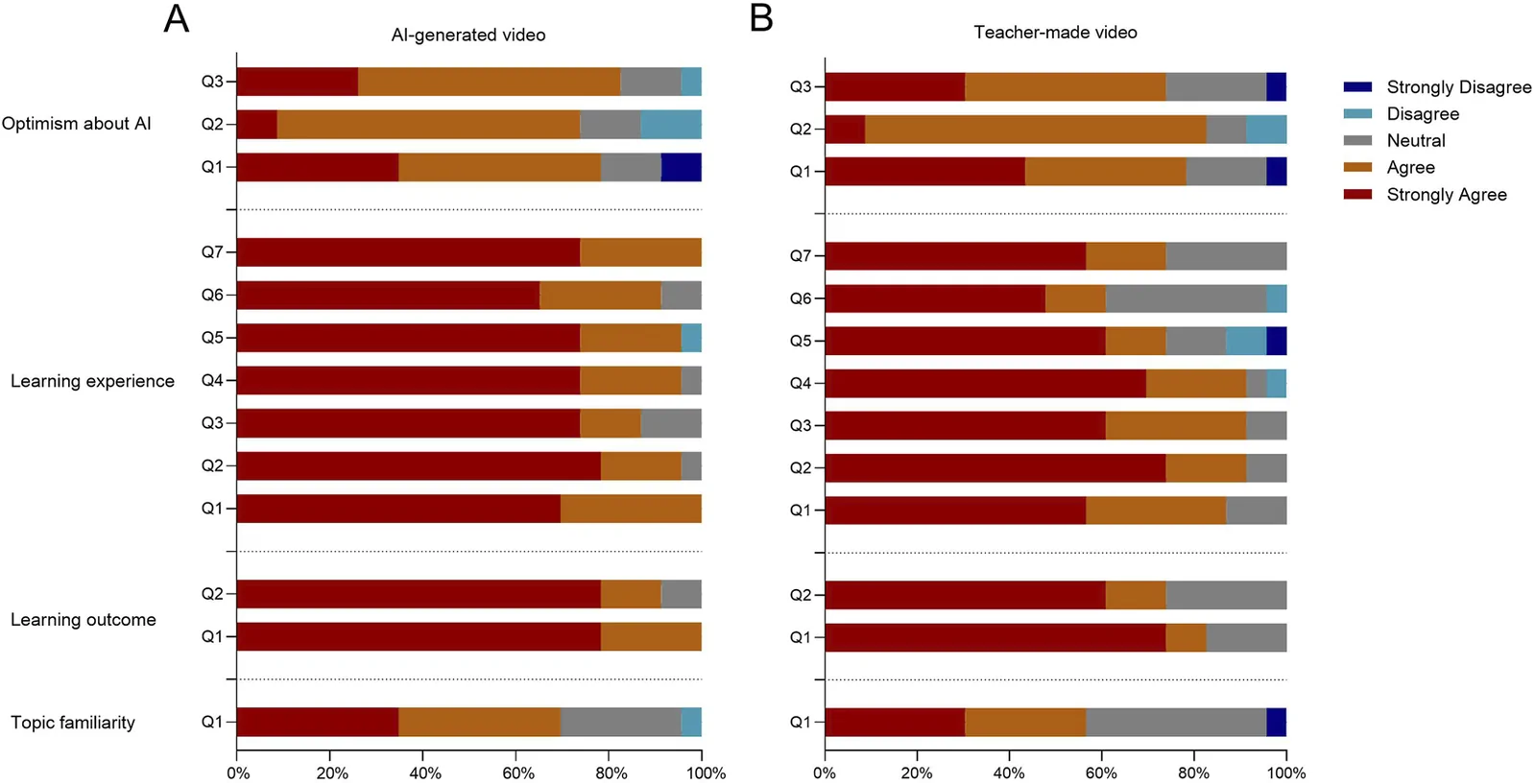
Results of the subjective questionnaires. **(A)** Evaluations of AI-assisted teaching video. **(B)** Evaluations of teacher-made teaching video.

**TABLE 4:** Descriptive statistics by AI-assisted vs. teacher-made videos, and their Cronbach alpha reliability coefficients (α).

Variable	Questions	Scores				α
		Total	AI-assisted	Teacher-made	p value	
Topic familiarity	1. I Know TED very well	3.89 ± 0.97	4.00 ± 0.90	3.78 ± 1.04	0.50	—
Learning outcome	1. I Have a deeper understanding of TED better after studying 2. I Am willing to recommend this teaching video to others	4.67 ± 0.63 4.52 ± 0.78	4.78 ± 0.42 4.70 ± 0.64	4.57 ± 0.79 4.35 ± 0.88	0.51 0.19	0.79
Learning experience	1. The overall quality of this video is high 2. TED core knowledge depth and difficulty suit well 3. The animations and illustrations are accurate and professional 4. The animations and illustrations are intuitive 5. The voiceover’s speed, clarity, and fluency are appropriate 6. The video is engaging, keeping me hooked throughout 7. The pacing is well-controlled, and the content flows logically	4.57 ± 0.62 4.70 ± 0.59 4.57 ± 0.69 4.63 ± 0.68 4.41 ± 1.02 4.30 ± 0.89 4.52 ± 0.72	4.70 ± 0.47 4.74 ± 0.54 4.61 ± 0.72 4.70 ± 0.56 4.65 ± 0.71 4.57 ± 0.66 4.74 ± 0.45	4.44 ± 0.72 4.65 ± 0.65 4.52 ± 0.67 4.57 ± 0.79 4.17 ± 1.23 4.04 ± 1.02 4.30 ± 0.88	0.27 0.78 0.52 0.72 0.22 0.08 0.10	0.90
Optimism about AI	1. I Often use AI tools to assist my learning 2. I Believe the content provided by AI tools is completely accurate 3. I Am willing to use AI-generated videos to learn about TED or other diseases in the future	4.11 ± 0.90 3.76 ± 0.77 4.00 ± 0.87	4.04 ± 0.93 3.70 ± 0.82 4.04 ± 0.77	4.17 ± 0.89 3.83 ± 0.72 3.96 ± 0.98	0.65 0.60 0.90	0.61

In the rating items, 1-Strongly Disagree, 2-Disagree, 3-Neither Agree nor Disagree, 4-Agree, 5-Strongly Agree. Statistically significant P values are in bold.

As shown in Table 5, correlation analysis across the dimensions revealed a strong positive correlation between learning experience and learning outcome (r = 0.60, p < 0.001). In contrast, topic familiarity showed no significant correlation with any other dimension.

**TABLE 5:** Spearman correlations between continuous and ordinal variables.

Variable	Topic familiarity	Learning outcome	Learning experience	Optimism about AI
Topic familiarity	1.00	​	​	​
Learning outcome	0.15	1.00	​	​
Learning experience	0.20	0.60***	1.00	​
Optimism about AI	−0.08	0.13	0.24	1.00

Coefficients close to 1 or -1 indicate a strong positive or negative correlation, respectively, while values closer to 0 suggest a weak or no correlation. Significance levels are reported as follows: ***p < 0.001, **p < 0.01, *p < 0.05.

We also collected residents’ responses to an optional question: “What is the aspect of the instructional video most in need of improvement?” Theme frequencies are reported descriptively in Supplementary Table S15. The most frequently mentioned themes were pacing (13/27, 48.1%), content comprehensiveness (9/27, 33.3%), visual quality (7/27, 25.9%), the respondents could mention multiple themes. As summarized in Table 6, the responses were categorized into three themes: content accuracy and structure; visual presentation and integrity; and overall quality. Feedback differed by video type: AI-assisted videos were criticized for stiff animation and insufficient highlighting of key terms, whereas teacher-made videos were noted for rigid visuals and narration, with labeling in need of improvement. Overall, the AI-assisted video was rated as high quality, while the teacher-made video was considered generally acceptable.

**TABLE 6:** Summary of medical practitioner feedback on medical instructional videos generated by the AI and teacher.

Video	Categories	Suggestions
AI-assisted	Content accuracy and structure	The video contained typographical errors The explanation of common TED symptoms in the second section was difficult to understand
	Visual presentation and integrity	The video lacked visual coherence and smooth frame transitions, and key terminology was not visually highlighted Many keywords appeared only in subtitles without corresponding visual cues. It is recommended to incorporate titles or keywords into on-screen visuals
	Overall quality	Pretty good
Teacher-made	Content accuracy and structure	Key points are not clearly highlighted with explanations. The instructional video should be structured around clinical practice applications
	Visual presentation and integrity	Animation effects are a bit abrupt The image could use more prominent, intuitive annotations The voiceover tone is somewhat rigid, with some pronunciation errors in disease terms Excessive speech rate and monotonous delivery negatively impacted learner engagement, necessitating appropriate pauses and more visual aids
	Overall quality	Overall acceptable

## 4 Discussion

### 4.1 Principal findings

Residency training is essential for developing competent physicians (Andrew G Lee and Carter, 2004). In China, standardized ophthalmology residency training was formally launched in 2010, much later than that in many developed countries (Wang et al., 2020). Although ophthalmology residency training lasts for 3 years, a significant gap remains in TED education, primarily due to a shortage of specialized educators. Given that DON is an ophthalmic emergency, timely diagnosis and intervention are essential to prevent irreversible vision loss.

To help address the educational gap, the present study explored the use of generative AI to develop a targeted instructional video on TED. Our goal was to preliminarily assess the feasibility and short-term learning outcomes of this AI-assisted video and to explore whether it would perform similarly to a teacher-made video. As general practitioners play a key role in early recognition, we included both ophthalmology and general residents, which also allowed us to explore its performance across different learner backgrounds.

Our findings revealed no statistically significant differences between the AI-assisted and teacher-made groups in short-term or long-term test score changes or completion time changes. Both groups showed significant score improvements at 5 min after learning, but these gains were not sustained at 1 week. The limited long-term retention may reflect the short video format and the lack of post-learning reinforcement. This suggests that short videos are best used as supplementary resources rather than primary teaching tools, and that students may benefit from additional review activities.

These findings are consistent with a previous study in which AI avatar-delivered Ear, Nose, and Throat (ENT) instructional videos—created using the HeyGen platform—significantly improved junior doctors’ confidence across multiple domains, with the greatest gains observed in recognizing endoscopic anatomy and triaging referrals (Siddique and Maweni, 2025). Another study also demonstrated that AI-assisted content achieves learning outcomes comparable to human-made videos, with the added benefits of faster production and lower cost (Netland et al., 2025).

Nevertheless, the limited efficacy in teaching DON may be partially attributable to the time constraint imposed by the video format. With the length restriction, the content on DON could only be covered briefly. Therefore, the findings highlight the need for a targeted video specifically for DON.

Exploratory subgroup analyses by learner background showed that after viewing the AI-assisted video, ophthalmology residents had numerically higher Test 3 scores, whereas general residents showed a numerically greater reduction in completion time at Test 3. These observations suggest possible differential patterns by residency type. However, because these subgroup differences were not formally tested, the potential utility of AI-assisted tools across learner backgrounds remains to be established.

Spearman correlation analysis revealed a strong positive correlation between learning experience and learning outcomes, indicating that more positive learning experiences are associated with better learning outcomes. However, no statistically significant correlation was observed between optimism about AI and learning outcomes. Future studies with larger samples and validated measures are needed to examine whether residents with less favorable attitudes can also benefit from the instructional tool.

Both the AI-assisted and teacher-made videos received common critiques, including excessively rapid speech delivery and insufficient focus in content presentation. These limitations may be attributable to the fact that both types of videos were limited to approximately 5 minutes in length, consistent with the format prescribed by micro-teaching principles (Nguyen and Diederich, 2023). Although this study did not address whether these findings extend to more complex instructional content or longer video formats, it provides a valuable reference for the field, highlighting the potential of AI tools as an aid in medical education (Netland et al., 2025).

Despite these shared constraints, the overall quality of the AI-assisted video was rated higher than that of the teacher-made video by participants, with 65% of participants agreeing that “The video is engaging, keeping me hooked throughout.” This observation is broadly consistent with previous research on AI-assisted videos (Contreras et al., 2024): 76% of the participants considered these videos very attractive, and 40% reported that their attention was well captured. With further advances in AI technology, future iterations may help reduce production time, but expert curation is likely to remain essential to ensure clinical accuracy and educational appropriateness.

Previous studies indicate that AI-assisted videos face challenges in terms of accuracy, reliability, and authenticity. As demonstrated by Han et al. (Han et al., 2024), even if the script content is generally correct, fictional ophthalmic imagery may pose risks related to visual authenticity, such as an operating lamp depicted as generating a laser beam. Similarly, a replication study conducted by Yaara et al. (Artsi et al., 2025) identified spelling errors when using the same prompts. Despite these limitations, most participants in the online experiments considered the teaching videos highly accurate in our study. This may be attributable to our rigorous screening and testing procedures.

Our findings suggest that no significant between-group differences were detected between AI-assisted and teacher-made videos in TED education. These findings may inform future research on generative-AI in other ophthalmic conditions and broader medical field (Lim et al., 2023; Darvishi et al., 2024). However, the AI-assisted workflow required substantial expert oversight and ultimately entailed greater human time costs. Given the limited long-term retention, such AI-assisted videos may be better positioned as supplementary resources combined with human expertise. We believe that AI-assisted tools have the potential to become valuable assistance tools in ophthalmology education if applied prudently and ethically (Kuang et al., 2025).

### 4.2 Future prospects

As AI moves toward practical use in medical education, healthcare institutions should develop systematic AI literacy and ethical training, as well as launching pilot educational initiatives. To ensure the safe, equitable and standardized implementation of AI-assisted teaching, the interdisciplinary supervision frameworks are required. Integrating feedback from clinical educators and residents into AI tool design and evaluation offers one potential pathway to balance clinical practicality, user accessibility, and long-term acceptance. Our findings may provide preliminary insights for institutions with similar digital infrastructure. However, given the substantial expert curation required, AI-assisted videos should be regarded as supplementary resources under expert supervision, and their feasibility in resource-limited settings remains to be determined. Future adequately powered studies are needed to evaluate whether such tools can enhance clinical training and reduce educational disparities.

### 4.3 Limitations

This study has several limitations. One of the key limitations is that the three knowledge-test forms were not formally equated, although they followed the same content blueprint and showed broadly comparable item-level difficulty and discrimination. Additionally, the absence of a no-video control group means that within-group score changes cannot be attributed to the educational videos; these changes should be interpreted as associations rather than causal effects.

In addition, the sample size was calculated for the primary within-group endpoint rather than for between-group differences. Consequently, the study lacked sufficient power to formally test equivalence or non-inferiority, and no non-inferiority margin was prespecified. The absence of a statistically significant between-group difference does not establish equivalence.

Moreover, the follow-up was limited to 1 week; long-term retention and durability of any learning effects remain unclear. Additionally, because the study was conducted online, limitations related to data quality and participant engagement cannot be excluded. Learning outcomes were assessed only through knowledge tests, not clinical performance. Future studies incorporating objective structured clinical examinations (OSCEs) or direct observation of clinical reasoning would provide stronger evidence.

Finally, although the AI-assisted content was reviewed by experts, AI-specific limitations such as factual hallucinations and undetected clinical inaccuracies were not systematically quantified. Future work should integrate rigorous automated validation to ensure clinical and factual integrity. Given the rapid advancement of AI, future versions may be more adaptive, personalized, and interactive, potentially surpassing traditional human-made materials. Larger, more diverse samples with extended follow-up are needed to confirm these exploratory findings.

## Data Availability

The datasets presented in this study can be found in online repositories. The names of the repository/repositories and accession number(s) can be found below: DOI: 10.6084/m9.figshare.33955012.
