# Supplementary material for: Comparing teacher-made and AI-assisted teaching videos for thyroid eye disease: an exploratory study
Source: Front Cell Dev Biol. 2026 Oct 21;14:1963967. doi: 10.3389/fcell.2026.1963967 (PMC13731330; doi:10.3389/fcell.2026.1963967)
Supplement: Supplementary file 1 [file Table1.docx]

Supplementary Material

**Note:** The questions and teaching materials in this Supplementary Material are reproduced as they were administered to participants during the study to preserve an accurate record of the educational intervention and assessments. Correct answers and explanatory materials were not visible to participants during any assessment and were released only after all study assessments had been completed. They are presented alongside the questions here solely to facilitate readers’ interpretation and evaluation of the assessment materials. Following peer review, all clinical questions, answer keys, explanations, and video statements in Supplementary Tables S1, S3, and S4 were independently re-reviewed against the 2021 EUGOGO clinical practice guideline and relevant region-specific sources. Where this post-study review identified statements requiring additional qualification or clarification, the original participant-facing content was retained for methodological transparency, and the corresponding additions are explicitly labeled as “Post-study author clarification.” These clarifications were added during manuscript revision and were not available to participants during the study.

**Supplementary Table S1.** Online experiment with the basic knowledge of Thyroid Eye Disease (TED).

Dear participants,

Thank you for participating in the experiment, which aims to assess trainees’ and junior residents’ baseline knowledge of thyroid eye disease during the ophthalmology rotation. The experiment is anonymous. All data collected will be used exclusively for this experiment and not for any other purposes.

Please complete the following questions **based on your current understanding, without recourse to online resources**. Your honest and complete responses are crucial to the validity of this research.

We greatly appreciate your time and invaluable assistance in this online experiment despite your busy schedule. Thank you for your cooperation!

The Research Team

Part 1. Personal information collection

Q1. Your Department and Position [Single-choice Question]

A. Ophthalmology Resident (Standardized Training)
B. General Resident (Standardized Training)

Q2. Your Gender [Single-choice Question]

A. Male
B. Female

Q3. Your Age: ___ years old. [Fill-in-the-blank]

Q4. Your initials: ___. (e.g., San Zhang: sz. It’s for distinguishing respondents only and will not disclose any personal information. Thank you for your cooperation!) [Fill-in-the-blank]

Part 2. Clinical questions about TED (including level of difficulty, answers and explanations).

(Difficult) Q1. What is the most frequently affected extraocular muscle in TED? [Single-choice Question]

A. Superior rectus
B. **Inferior rectus (Answer)**
C. Medial rectus
D. Lateral rectus

Explanation: The typical order of extraocular muscle involvement in TED is inferior rectus > medial rectus > superior rectus > lateral rectus.

(Moderate) Q2. What is the most common cause of eyelid retraction in Thyroid Eye Disease? [Single-choice Question]

A. **Fibrosis of the levator palpebrae superioris (Answer)**
B. Levator aponeurosis disinsertion/tear
C. Orbicularis oculi muscle spasm
D. Skin laxity

Explanation: In TED, congestion and edema of the levator muscle can lead to subsequent fibrosis, increasing its tone and pulling the eyelid upward, resulting in retraction.

(Easy) Q3. What is the most valuable imaging modality for diagnosing Thyroid Eye Disease? [Single-choice Question]

A. X-ray plain film
B. B-scan ultrasonography
C. **Orbital CT (coronal view) (Answer)**
D. Cerebral angiography

Explanation: Orbital CT can clearly demonstrate enlargement of the extraocular muscle bellies with relative sparing of the tendons.

(Easy) Q4. Regarding the impact of smoking on Thyroid Eye Disease, which statement is INCORRECT? [Single-choice Question]

A. Smoking is the most significant environmental risk factor for the development and progression of TED.
B. Smoking reduces the efficacy of glucocorticoid therapy and orbital radiotherapy.
C. **Smoking primarily affects the severity of TED but is not associated with disease activity. (Answer)**
D. Advising patients to quit smoking is a crucial part of basic TED management.

Explanation: Smoking not only exacerbates the severity of TED but is also associated with higher disease activity and poorer response to treatment.

(Difficult) Q5. What is the most characteristic sign of the active phase of Thyroid Eye Disease? [Single-choice Question]

A. Proptosis
B. Eyelid retraction
C. Restriction of eye movements
D. **Conjunctival congestion and edema (Answer)**

Explanation: The active phase is characterized primarily by inflammatory signs, including conjunctival congestion, edema, and caruncular edema. Other options are more typical of chronic-phase changes, such as fibrosis leading to motility restriction.

(Moderate) Q6. What is the “gold standard” for assessing the activity of Thyroid Eye Disease? [Single-choice Question]

A. **Clinical Activity Score (CAS) (Answer)**
B. Thyroid function tests (T3, T4, TSH)
C. Signal intensity on T2-weighted orbital MRI
D. Patient-reported symptom scores

Explanation: The CAS is the most commonly used and convenient tool for assessing disease activity in clinical practice.

**Post-study author clarification**: The term “gold standard” in the original assessment item was overly categorical. The 2021 EUGOGO guideline describes the Clinical Activity Score (CAS) as the best-validated scoring system for assessing disease activity, while acknowledging its limitations. The original wording has been retained here to accurately reproduce the assessment administered to participants.

(Moderate) Q7. What is NOT a component of the Clinical Activity Score (CAS)? [Single-choice Question]

A. Conjunctival injection (redness)
B. Eyelid erythema or edema
C. **Proptosis > 20 mm (Answer)**
D. Spontaneous retrobulbar pain

Explanation: Proptosis is an indicator of disease severity, not activity.

(Easy) Q8. A patient with thyroid-associated ophthalmopathy presents with visual field defects and decreased color vision. The most likely cause is? [Single-choice Question]

A. Retinal detachment
B. **Dysthyroid optic neuropathy (DON) (Answer)**
C. Optic neuritis
D. Corneal ulcer

Explanation: DON, primarily caused by compression of the optic nerve, presents with decreased visual acuity, visual field defects, and impaired color vision.

(Moderate) Q9. An 8-year-old boy presents with acute pain, redness, and swelling of the right eyelid for 2 days, which is worsening. Examination: Temperature 39°C, marked redness, swelling, and warmth of the right eyelid with significant tenderness. Conjunctival congestion and chemosis, mild proptosis, and restricted eye movements are noted. The most likely diagnosis is? [Single-choice Question]

A. Orbital inflammatory pseudotumor

B. Orbital rhabdomyosarcoma

C. Orbital chloroma (Granulocytic sarcoma)

D. **Orbital cellulitis (Answer)**

Explanation: Orbital cellulitis is more common in children than adults, presents acutely with painful eyelid swelling, proptosis, and often systemic symptoms like fever. TED typically occurs in patients aged 30-50, more often in females, with thyroid dysfunction but without fever. CT and blood tests can aid in differentiation.

(Moderate) Q10. What is a characteristic feature of orbital CT that assists in differentiating Thyroid Eye Disease from orbital inflammatory pseudotumor? [Single-choice Question]

A. **Enlargement of the muscle belly with relative sparing of the tendon (Answer)**

B. Enlargement of extraocular muscles

C. Increased density of orbital fat

D. Enlargement of the superior ophthalmic vein

Explanation: In myositis orbital pseudotumor, the muscle is enlarged, but typically involves both the tendon and belly unilaterally. TED more commonly involves multiple muscles bilaterally, with enlargement of the muscle bellies, while the tendons appear normal.

(Easy) Q11. What is the first-line management for mild, inactive Thyroid Eye Disease? [Single-choice Question]

A. Glucocorticoid therapy

B. Radiotherapy

C. **Supportive and conservative management (e.g., artificial tears) (Answer)**

D. Surgical intervention

Explanation: For mild, inactive TED, common management includes observation, risk factor control, and symptomatic supportive treatment. Surgery may be considered later for residual appearance-related issues if necessary.

(Easy) Q12. What is the first-line treatment for moderate-to-severe, active Thyroid Eye Disease? [Single-choice Question]

A. **High-dose intravenous glucocorticoid pulse therapy (Answer)**

B. Immediate orbital decompression surgery

C. Oral non-steroidal anti-inflammatory drugs (NSAIDs)

D. Supportive treatment only (e.g., artificial tears)

Explanation: For moderate-to-severe, active TED, the first-line treatment is intravenous glucocorticoid pulse therapy. The classical regimen involves a cumulative dose of 4.5g (e.g., 0.5 g weekly for 6 weeks + 0.25 g weekly for 6 weeks).

(Difficult) Q13. Which biological agent is considered an off-label use for the treatment of TED in China? [Single-choice Question]

A. Teprotumumab

B. Both are off-label

C. Neither is off-label

D. **Tocilizumab (Answer)**

Explanation: According to the consensus, Teprotumumab has been approved for TED, while the use of Tocilizumab and Rituximab for TED treatment in China is considered off-label.

**Post-study author clarification**: Drug approval and off-label status are region- and time-specific. The regulatory status of these biologic agents was re-verified for mainland China in relation to the timing of the present educational experiment. Teprotumumab N01 had received regulatory approval for TED in mainland China before the study assessments were conducted, whereas TED was not an approved indication for tocilizumab in mainland China; therefore, its use for TED remained off-label. The relevant region-specific regulatory sources and verification date have now been added.

(Moderate) Q14. Among the following examinations for patients with Thyroid Eye Disease, which is performed LEAST frequently? [Single-choice Question]

A. Corneal staining

B. **OCT (Optical Coherence Tomography) (Answer)**

C. Ocular motility examination

D. Exophthalmometry

Explanation: In the evaluation of TED, corneal staining assesses exposure keratopathy, ocular motility examination assesses extraocular muscle function, and exophthalmometry measures proptosis. OCT is not a routine or primary investigation for standard TED assessment.

(Difficult) Q15. For patients with active TED who are unresponsive or intolerant to glucocorticoid therapy, which of the following is NOT considered a second-line treatment option? [Single-choice Question]

A. Orbital radiotherapy

B. Immunosuppressants (e.g., cyclosporine, mycophenolate)

C. Biological agents (e.g., teprotumumab, rituximab)

D. **Strabismus surgery (Answer)**

Explanation: To correct residual misalignment, strabismus surgery is a rehabilitative procedure during the inactive phase. The second-line treatments for active TED include immunosuppressants, biological agents, and orbital radiotherapy.

(Easy) Q16. Regarding the management of restrictive strabismus caused by Thyroid Eye Disease, what is the correct sequence of interventions? [Single-choice Question]

A. Perform strabismus surgery first, then decompression surgery
B. **Perform orbital decompression surgery first, and then consider strabismus surgery after stabilization (Answer)**
C. Perform only strabismus surgery
D. Strabismus and decompression surgery can be performed simultaneously

Explanation: The principle for the sequence of multiple surgeries in TED is that decompression surgery can alter eye position; therefore, it must be performed before strabismus surgery.

(Moderate) Q17. There’s a 45-year-old female with a 6-month history of treated Graves’ disease (euthyroid on medication), presenting with 1 month of bilateral eye pain, tearing, and diplopia. Examination results: Bilateral eyelid erythema and edema, marked conjunctival congestion and chemosis. Proptosis: 20mm OD (base 100mm) 19mm OS. Restricted eye movements in all directions. CAS=5. Visual acuity, color vision, and fundus exam are unremarkable. What is the first-line treatment? [Single-choice Question]

A. Immediate orbital decompression

B. Oral thyroxine

C. Observation with supportive care only

**D. Intravenous high-dose glucocorticoid pulse therapy (Answer)**

Explanation: The patient presents with active, moderate-to-severe TED, and the first-line treatment is intravenous glucocorticoid pulse therapy.

(Easy) Q18. There’s a 60-year-old male with weight loss, diaphoresis, and polyphagia. Laboratory tests showed elevated T3 and T4 and low TSH. Subsequently, he developed bilateral proptosis and horizontal binocular diplopia. Examination results: Bilateral upper eyelid edema. The upper lid margin is at the superior limbus, and the lower lid margin is at the inferior limbus. Eyelid closure is complete. Significant limitation of abduction bilaterally, worse in the right eye. Based on the clinical description and provided images (CT showing bilateral proptosis and enlarged extraocular muscles), the initial diagnosis is: [Single-choice Question]

**A. Thyroid Eye Disease (TED) (Answer)**

B. Inflammatory pseudotumor

C. Orbital cellulitis

D. Simple endemic goiter

Explanation: The patient has a history of hyperthyroidism, with subsequent proptosis, diplopia, upper eyelid retraction, and restricted eye movements. CT findings of bilateral proptosis and enlarged extraocular muscles are consistent with TED.

(Difficult) Q19. A 38-year-old female with active TED (CAS=6) received one cycle of intravenous injection of glucocorticoid pulse therapy. There is partial improvement in diplopia and inflammation, but she has persistent proptosis and develops dysthyroid optic neuropathy (DON) with visual acuity of 0.3. What is the most appropriate next step? [Single-choice Question]

A. Continue with a second cycle of glucocorticoid pulses

**B. Urgent orbital decompression surgery (Answer)**

C. Eyelid retraction repair surgery

D. Observation

Explanation: While intravenous injection of glucocorticoids is first-line for DON, urgent surgical decompression is indicated for patients with DON, especially those who are unresponsive or only partially responsive to medical therapy, to relieve optic nerve compression.

(Difficult) Q20. A 48-year-old male with a history of untreated hyperthyroidism presents with a 1-week history of decreased vision in the left eye. Examination results: Left eye: CAS=5; visual acuity is counting fingers at 10cm (no improvement with pinhole); decreased sensitivity in the superior visual field; IOP: 21 mmHg. Orbital CT is shown (suggesting apical crowding). The most urgent management is: [Single-choice Question]

A. High-dose oral prednisone

B. Emergent lateral canthotomy and cantholysis

**C. Intravenous glucocorticoid pulse therapy（Answer）**

D. Schedule orbital decompression surgery for next week

Explanation: The patient with active TED and acute vision loss is likely to have DON, an ophthalmic emergency. Upon diagnosis, immediate high-dose intravenous glucocorticoid pulse therapy should be initiated for medical decompression.

**Supplementary Table S2.** Example of an AI-generated video by the generative AI.

Step 1. Script generation with Deepseek

Step 2. Visuals generation with Dreamina AI.

Step 3. Videos generation with CapCut tool.

Step 1. Script generation with Deepseek

Video Synthesis Prompt for Dreamina AI platform: Core Clinical Insights into Thyroid Eye Disease

Video Title: Core Clinical Insights into Thyroid Eye Disease

Video Style: A combination of professional medical animation and a virtual lecture hall. The style is rigorous and transparent, supplemented by high-quality 3D medical animations, diagrams, and limited clinical imagery, focusing purely on knowledge transfer.

Target Audience: Ophthalmology residents, General residents.

Core Objective: To systematically consolidate key knowledge points of Thyroid Eye Disease (TED), enhancing the understanding of its pathophysiology, diagnostic evaluation, and treatment strategies.

Video Duration: Approximately 4-5 minutes.

Video Scene Breakdown and Prompts

**[Opening: Overview]**

-Visuals: A young male virtual lecturer stands at the center of a simply decorated studio, upright and poised, with a focused and confident gaze. His right hand is slightly raised, palm open naturally, as if explaining a concept to the audience. Behind him, a large screen clearly displays the title “Thyroid-Associated Ophthalmopathy,” accompanied by detailed anatomical diagrams that illustrate the structures of the orbit and extraocular muscles. The overall scene conveys an atmosphere of professionalism and focus.

-Narration: “Thyroid Eye Disease (TED) is an organ-specific autoimmune disorder. A deep understanding of its core principles is fundamental to accurate diagnosis and treatment. This video will focus on several key clinical aspects.”

**[Part 1: Pathogenesis and Characteristic Manifestations]**

-Key Knowledge 1: Pathogenesis (TRAb, IGF-1R), Pathological Changes (Inflammation, GAG Deposition).

-Visuals: An image depicting the microscopic cellular level in a patient with thyroid-associated ophthalmopathy shows numerous TRAb-labeled antibodies above the orbital fibroblasts, resembling keys. The surface of the fibroblasts is covered with IGF1R and TSHR receptors, depicted as locks. Some of the TRAb antibodies are shown fitting into these receptor locks, like keys into their corresponding keyholes. Then a variety of cytokines emanate from the upper left region of the cell, forming an elliptical band labeled with TNF-α (white), IFN-γ (yellow), IL-6 (purple), and others. Meanwhile, numerous spherical glycosaminoglycans (GAGs) (green) drift from the upper right side of the cell, with their color transitioning from blue to red. Furthermore, a close-up view reveals numerous swollen cells with a vivid red appearance. Each cell is distinctly visible, as if under high magnification, exhibiting detailed and unique morphological features. Extensive cellular swelling and proliferation ultimately lead to expansion of the muscle and fat tissues within the orbit.

-Narration: “The instigator of TED is an autoantibody known as the TSH receptor antibody, or TRAb. Acting like an incorrect key, it not only targets the thyroid but also synergizes with IGF‑1R on orbital fibroblasts. This dual signal activates the cells, causing them to overproduce and secrete glycosaminoglycans. GAGs act like sponges, absorbing up to 100 times their own weight in water, leading to severe edema and expansion of tissues and muscles, akin to gradually inflating a water balloon. This is the fundamental pathological root of all clinical manifestations of TED.”

-Key Knowledge 2: Pattern of Extraocular Muscle Involvement

-Visuals: Presented as high-quality 3D images, these visuals provide a macroscopic view of the face and clearly depict the interior of the orbit in a patient with thyroid-associated ophthalmopathy, revealing thickened extraocular muscles and swollen orbital fat.

-Narration: “TED primarily involves the extraocular muscles, and its involvement follows a characteristic order: the inferior rectus is most frequently affected, followed by the medial rectus, superior rectus, and lateral rectus.”

-Key Knowledge 3: Mechanism of Eyelid Retraction

-Visuals: This 3D animation clearly illustrates the four extraocular muscles—the superior, inferior, medial, and lateral rectus—within the orbit. The left side depicts a normal orbit, while the right side shows an orbit affected by Graves' ophthalmopathy, with particular focus on the inferior and medial rectus muscles. Animation focuses on the levator palpebrae superioris muscle, demonstrating its inflammation, edema, and subsequent fibrosis and contracture. It dynamically shows how this process elevates the eyelid, widening the palpebral fissure.

-Narration: “Eyelid retraction is a hallmark sign of TED. The core mechanism is fibrosis of the levator muscle, leading to increased muscle tension, rather than simple sympathetic nerve overstimulation.”

**[Part 2: Clinical Manifestations and Diagnosis]**

-Key Knowledge 1: Symptoms (Foreign Body Sensation, Diplopia), Signs (Eyelid Retraction, Proptosis, Conjunctival Injection), CAS Components.

-Visuals: A series of cinematic vignettes depicting a patient with TED (young to middle-aged female) from early to middle stages. 1. A young woman with a pained expression is shown working at a computer, frequently blinking and rubbing her eyes. The text "foreign body sensation" appears in a circle adjacent to her. 2. A scene from a clinical examination: the doctor shines a light into the young woman's eyes. A close-up view reveals marked vascular congestion and edema of the bulbar conjunctiva and lacrimal caruncle. The image is labeled with the text "Conjunctival congestion, lacrimal caruncle edema." 3. A close-up of a young woman with a terrified expression. As she looks in the mirror, her palpebral fissures are noticeably widened, giving her eyes a "staring" appearance. The text "eyelid retraction" is clearly circled adjacent to the image. 4. A young woman experiences double vision while walking down stairs, causing her to nearly fall and reach out to steady herself against the wall. The text "double vision" is circled adjacent to the image. 5. The doctor guides the patient's eye movements by moving a light source upward and then from left to right.

-Narration: “When the immune storm hits, the eyes send clear distress signals. Patients often first experience dryness, a foreign-body sensation, and photophobia, which are caused by incomplete eyelid closure and inflammation. The most characteristic signs are eyelid retraction, giving the patient a ‘frightened’ appearance, and proptosis. As extraocular muscles become involved, patients develop diplopia, also known as double vision, which can be either vertical or horizontal. Vertical diplopia is more common, corresponding to the fact that the inferior rectus muscle is most frequently affected. The gold standard for assessing disease ‘activity’ is the Clinical Activity Score. Please remember these seven inflammatory signs: spontaneous orbital pain, gaze-evoked pain, eyelid erythema, conjunctival injection, conjunctival chemosis, caruncle edema, and eyelid edema. A CAS score of 3 or more indicates active, progressive disease.”

**Post-study author clarification**: “Gold standard” should be interpreted here as referring to CAS as the best-validated clinical scoring system for disease activity according to the 2021 EUGOGO guideline, rather than as an absolute diagnostic gold standard.

-Key Knowledge 2: Role of Orbital CT/MRI, CAS.

-Visuals: A split-screen video. The image is divided into two panels: a CT model of the orbit (left) and a clinical examination scene (right).. On the right, The image depicts a clinical examination scenario in which a doctor performs a CAS (Clinical Activity Score) assessment on a patient. This includes applying light pressure to the eyeball to elicit spontaneous pain, observing conjunctival injection and eyelid edema, and asking the patient to look upward to assess for eyelid lag.

-Narration: “Orbital CT or MRI is the 'revealing mirror' for diagnosing TED. The most common imaging feature is fusiform or spindle-shaped enlargement of the muscle bellies of multiple extraocular muscles, sparing the muscle tendons. Please note, this is a key differentiating point from conditions like orbital inflammatory pseudotumor. The frequency of muscle involvement is: inferior rectus, medial rectus, superior rectus, and lateral rectus. These enlarged muscles occupy the limited space within the orbit, which pushes the eyeball forward, leading to proptosis, and in severe cases, can compress the optic nerve, threatening vision.”

**[Part 3: Core Dimensions of Disease Assessment]**

-Key Knowledge 1: Distinction Between Activity and Severity

-Visuals: The image displays the words "Mobility vs. Severity" in large letters against a black background, with the text in white and "vs" highlighted in red. Then screen split into left and right. Left side lists “Activity” with dynamic footage of signs like conjunctival injection, caruncle edema, eyelid erythema. Right side lists “Severity” with diagrams or images of static signs, such as proptosis, eyelid retraction, and diplopia.

-Narration: “Distinguishing between disease activity and severity is the cornerstone of formulating a treatment plan. Activity reflects the current level of inflammation, commonly assessed using the Clinical Activity Score. Severity represents the degree of structural damage caused by the disease. These two concepts are distinct and determine whether we should employ immuno-suppressive therapy or consider rehabilitative surgery.”

-Key Knowledge 2: Dysthyroid Optic Neuropathy (DON)-An Ophthalmic Emergency

-Visuals: A dynamic illustration depicting optic nerve compression in thyroid-associated ophthalmopathy. The extraocular muscles at the orbital apex are markedly thickened, creating a narrow "bottleneck" that compresses the optic nerve. The image also clearly demonstrates associated visual field defects and abnormalities on color vision testing. The overall composition is richly detailed and visually compelling.

-Narration: “Dysthyroid optic neuropathy is the most severe complication of TED and constitutes an ophthalmic emergency. It results from compression of the optic nerve by enlarged extraocular muscles at the orbital apex. Clinically, it presents with decreased visual acuity, color vision deficits, and visual field defects. Early recognition and urgent intervention are critical. Once DON is diagnosed, prompt initiation of high-dose corticosteroid therapy is recommended. If no response to hormone therapy is observed within two weeks, emergency orbital decompression should be performed.”

**Post-study author clarification**: The timing in the original narration was simplified. According to the 2021 EUGOGO guideline, DON should be treated immediately with high-dose intravenous methylprednisolone, and urgent orbital decompression should be performed if the response is absent or poor within 1–2 weeks. Urgent surgical escalation is also required when visual function deteriorates despite medical treatment.

**[Part 4: Stepped Management of Treatment Strategies]**

-Key Knowledge 1: Principles of Graded Treatment

-Visuals: A clear, stepped treatment pathway diagram unfolds dynamically.

- Step 1: “Mild, Inactive” -> Animation shows icons for artificial tears, smoking cessation counseling, and controlling thyroid function.

- Step 2: “Moderate-Severe, Active” -> Highlights “Intravenous Glucocorticoid Pulse Therapy” as the first-line option.

- Step 3: “Steroid-Refractory or Contraindicated” -> Introduces second-line choices like “Orbital Radiotherapy”, “Immunosuppressants”, and “Biologics”.

- Step 4: “Stable, Inactive” -> Displays animations of rehabilitative surgeries: “Orbital Decompression”, “Strabismus Surgery”, “Eyelid Surgery”.

-Narration: “TED treatment follows stepped and individualized principles. The treatment tier must be chosen based on the disease's activity and severity. Mild and inactive TED, are mainly treated with artificial tears, smoking cessation counseling and controlling thyroid function. Moderate-severe and active TED, intravenous glucocorticoid pulse therapy is the first line of treatment. When hormones are refractory or contraindicated, second-line treatment may be considered, such as, orbital radiotherapy, immunosuppressants and biologics, etc. Only in stable and inactive TED, the restorative surgery is typically considered. Certainly, emergency decompression surgery for DON should be excluded.”

**Post-study author clarification**: This statement incompletely describes the 2021 EUGOGO first-line recommendation. For most patients with moderate-to-severe, active TED, the guideline recommends intravenous methylprednisolone combined with mycophenolate sodium (or mofetil) as first-line treatment. Intravenous methylprednisolone monotherapy at a higher cumulative dose may be considered in selected more severe cases.

-Key Knowledge 2: The Irreversible Sequence of Surgeries

-Visuals: Animation sequentially demonstrates the three steps: Orbital Decompression Surgery (expanding the bony orbit), Extraocular Muscle Surgery (correcting strabismus), and Eyelid Surgery (correcting retraction), clearly labeled with numbers 1, 2, and 3.

-Narration: “If multidisciplinary surgery is required, a strict order must be followed: first, orbital decompression; second, strabismus surgery to correct eye misalignment; and finally, surgery to correct eyelid retraction. This sequence should not be reversed, as each preceding step alters the anatomical relationships for subsequent procedures.”

-Key Knowledge 3: Mechanism of Action of Teprotumumab (IGF-1R Ab) and Tocilizumab (IL-6R Ab).

-Visuals: 1.The main focus of the image is a thickened, spindle-shaped extraocular muscle, whose tissue is composed of clusters of fibroblasts. A white, Y-shaped teprotumumab antibody is shown binding specifically to the IGF-1R target on the fibroblast surface. The GAG molecule secreted by the cell is depicted with a cross, indicating the inhibition of its secretion, which subsequently leads to cell atrophy. 2. Numerous IL-6 molecules are shown hovering above the fibroblasts, attempting to bind to their receptors on the cell surface. Meanwhile, a white Y-shaped antibody labeled "tocilizumab" has already bound to a receptor, blocking it like a shield.

-Narration: “When traditional treatments yield insufficient results, we now have precise weapons: biologics.

- Teprotumumab acts like a specially crafted key, precisely locking onto the ‘IGF-1 receptor’ target on orbital cells. Upon binding, it directly blocks the key signaling pathway responsible for tissue expansion and edema, effectively causing the pathological cells to 'cease work' at the source, thereby rapidly alleviating symptoms like proptosis.

- Tocilizumab, on the other hand, is a highly efficient ‘firefighter’. It specifically extinguishes the ‘fire’ ignited by potent inflammatory factors, such as ‘IL-6’. By blocking the binding of IL-6 to its receptor, it rapidly subsides the inflammatory storm within the orbit, particularly suitable for active-phase patients who are refractory to corticosteroids.

- These two drugs, one curbing the disease at the source of ‘growth’ and the other extinguishing the ‘inflammation’ link, together represent the arrival of a new targeted era in the treatment of TED.”

**[Conclusion: Summary and Integration]**

-Visuals: The virtual lecturer returns to the center of the screen. The screen behind rapidly reviews core keyword concepts from the video (e.g., Muscle belly enlargement, Activity/Severity, DON, Stepped treatment, Surgical sequence).

-Narration: “Mastering the pathological features of TED, learning to conduct accurate assessments, and following a scientific treatment pathway are key to providing optimal care for our patients. We hope this core insights session helps you build a clear framework for clinical thinking.”

Step 2. Visuals generation with Dreamina AI.

The output visual illustrations often exhibited notable variability and occasional inappropriateness, necessitating more careful screening and guidance compared to the text content generated by DeepSeek. To ensure clinical and pedagogical quality, all AI-generated visual content underwent a rigorous expert review process. The review panel comprised two senior ophthalmologists with expertise in thyroid eye disease (TED) and one medical education specialist, who evaluated the content across three dimensions: clinical accuracy (alignment with current guidelines and evidence-based knowledge), pedagogical appropriateness (clarity, logical structure, and suitability for residency training), and visual reliability (absence of misleading or fictional imagery). Any content identified as inaccurate or potentially misleading was flagged for revision, and the AI system was prompted to regenerate the relevant segments. The revised content was then re‑evaluated by the same panel. This iterative process continued until all reviewers reached a consensus that the final version met the required standards. No major factual errors were identified after the final round of review.

Step 3. Videos generation with CapCut tool.

The core workflow comprises four sequential stages: importing source materials, arranging them on the timeline, applying edits and supplementary elements via the tools accessible from the top or right panels, and exporting the final output.

**Supplementary S3.** Online experiment with AI-teaching survey and exam questions.

Dear participants,

Thank you for participating in the online experiment, which aims to assess the learning effects of teaching videos on thyroid eye disease among trainees and junior residents during the ophthalmology rotation. After watching a five-minute teaching video, please rate your learning experience. The rating scale is 5 points, from “strongly disagree” to “strongly agree”.

The experiment is anonymous. All data collected will be used exclusively for this experiment and not for any other purposes.

We greatly appreciate your time and invaluable assistance in this online experiment despite your busy schedule. Thank you for your cooperation!

The Research Team

Part 1. Personal information collection

Q1. Your Department and Position [Single-choice Question]

A. Ophthalmology Resident (Standardized Training)
B. General Resident (Standardized Training)

Q2. Your Gender [Single-choice Question]

A. Male
B. Female

Q3. Your Age: ___ years old. [Fill-in-the-blank]

Q4. Your initials: ___. (e.g., San Zhang: sz. It’s for distinguishing respondents only and will not disclose any personal information. Thank you for your cooperation!) [Fill-in-the-blank]

Part 2. Videos and learning experience questions

Please rate the following statements about the teaching video.

[1-Strongly Disagree, 2-Disagree, 3-Neither Agree nor Disagree, 4-Agree, 5-Strongly Agree]

Q1. I know TED very well.

Q2. The overall quality of this video is high.

Q3. TED core knowledge depth and difficulty suit well.

Q4. The animations and illustrations are accurate and professional.

Q5. The animations and illustrations are intuitive.

Q6. The voiceover’s speed, clarity, and fluency are appropriate.

Q7. The video is engaging, keeping me hooked throughout.

Q8. The pacing is well-controlled, and the content flows logically.

Q9. I have a deeper understanding of TED better after studying.

Q10. I am willing to recommend this teaching video to others.

Part 3: AI Tool Usage Preferences

Please rate the following statements about AI tools.

[1-Strongly Disagree, 2-Disagree, 3-Neither Agree nor Disagree, 4-Agree, 5-Strongly Agree]

Q1. I often use AI tools to assist my learning.

Q2. I believe the content provided by AI tools is completely accurate.

Q3. I am willing to use AI-generated videos to learn about TED or other diseases in the future.

Q4. What is the aspect of the instructional video most in need of improvement? [Fill-in-the-blank, optional]

Part 4. Clinical questions about TED (including answers and explanations).

Finally, there’s a short test to assess the instructional video’s learning effectiveness. Please complete the following questions **based on your current understanding after reading the questions and answers effectively, without recourse to online resources**. Your honest and complete responses are crucial to the validity of this research. Thank you for your cooperation!

(Easy) Q1. 1. What is the primary pathological change in TED? [Single Choice]

A. Acute necrosis of orbital fat.
B. Fibrosis and contracture of extraocular muscles.
C. **Lymphocytic infiltration and glycosaminoglycan deposition in orbital tissues and extraocular muscles. (Answer)**
D. Atherosclerosis of orbital blood vessels.

Explanation: TED is an autoimmune inflammation, and its pathological changes are mainly: (1) orbital inflammatory lymphocyte infiltration. (2) New adipogenesis leads to the expansion of adipose tissue in extraocular muscle connective tissue and other orbital fat/connective tissue. (3) Orbital fibroblasts (OF) up-regulated the synthesis of hydrophilic glycosaminoglycans (GAG, mainly hyaluronic acid, HA), resulting in edema and further expansion of various orbital tissues. (4) OF can also differentiate into myofibroblasts, mediate extracellular matrix deposition, and eventually lead to tissue fibrosis.

(Easy) Q2. The pathogenesis of TED is most closely associated with which of the following autoantibodies? [Single Choice]

A. Thyroid Peroxidase Antibody (TPOAb)
B. **Thyrotropin Receptor Antibody (TRAb) (Answer)**
C. Thyroglobulin Antibody (TgAb)
D. Acetylcholine Receptor Antibody

Explanation: The core pathogenesis of TED: TSHR is the main autoimmune antigen in TED. TSHR stimulation of TRAb can activate cAMP production and pAKT level in orbital fibroblasts, and further promote the production of adipogenic factors and hyaluronic acid in orbital fibroblasts. Hyaluronic acid swelling can lead to edema of the extraocular muscles and orbital adipose connective tissue, increase the volume of orbital adipose tissue, and ultimately result in increased intraocular pressure and exophthalmos.

(Difficult) Q3. Which of the following is the most common ocular symptom of TED? [Single Choice]

A. Severe eye pain

B. Diplopia

C. Vision loss

D. **Foreign body sensation, dryness, and photophobia (Answer)**

Explanation: The common symptoms of TED include ocular irritation, such as ocular foreign body sensation, dryness, and photophobia. Diplopia and visual loss are serious complications.

(Moderate) Q4. In China, what are the normal reference ranges for proptosis? [Single Choice]

A. **12-14mm (Answer)**
B. 14-18mm
C. 18-22mm
D. >22mm

Explanation: The normal average proptosis value in Chinese people is 12-14mm.

(Easy) Q5. Which of the following eyelid signs is a classic manifestation of TED? [Single Choice]

A. Exophthalmos
B. **Eyelid retraction (Dalrymple’s sign) (Answer)**
C. Blepharospasm
D. Epicanthal fold

Explanation: Eyelid retraction is the most common symptom in patients with TED, and upper eyelid retraction is more common.

(Moderate) Q6. On orbital CT, what is the characteristic feature of extraocular muscle enlargement in typical TED? [Single Choice]

A. Thickening at the muscle insertion point
B. **Fusiform enlargement of the muscle belly with sparing of the tendon (Answer)**
C. Diffuse, uniform thickening of the entire muscle
D. Calcification within the muscle

Explanation: The characteristic imaging manifestation of TED is spindle-shaped muscle belly hypertrophy, and the tendon is usually not involved.

(Difficult) Q7. What is the most important investigation for diagnosing dysthyroid optic neuropathy (DON)? [Single Choice]

A. Exophthalmos measurement

B. Intraocular pressure measurement

**C.** **Assessment of visual acuity, color vision, and visual fields (Answer)**

D. Corneal fluorescein staining

Explanation: The clinical diagnosis of DON includes decreased vision, increased intraocular pressure, decreased color vision, decreased contrast sensitivity, positive relative afferent pupillary disorder (RAPD), optic disc changes, visual field defects, and abnormal visual evoked potential (VEP). At present, there is no uniform, clear diagnostic criterion for DON, but there is no doubt that it should be combined with imaging and clinical diagnosis.

(Difficult) Q8. The mechanism of action of Teprotumumab primarily involves blocking which receptor? [Single Choice]

A. **Insulin-like Growth Factor-1 Receptor (IGF-1R) (Answer)**
B. Interleukin-6 Receptor (IL-6R)
C. CD20 Receptor
D. Thyrotropin Receptor (TSHR)

Explanation: Teprotumumab is an IGF-1R monoclonal antibody that inhibits inflammation and fibrosis of orbital tissue from the source by accurately blocking the IGF-1R signaling pathway.

(Moderate) Q9. Which of the following conditions is NOT included in the differential diagnosis of TED? [Single Choice]

A. Orbital inflammatory pseudotumor
B. Carotid-cavernous fistula
C. Orbital myositis
D. **Age-related cataract (Answer)**

Explanation: Cataract is a lens disease that does not cause exophthalmos or extraocular muscle changes.

(Difficult) Q10. A patient presents with unilateral proptosis. Orbital CT shows uniform thickening of both the muscle belly and tendon of an extraocular muscle. What is the most likely diagnosis? [Single Choice]

A. Thyroid Eye Disease
B. **Orbital inflammatory pseudotumor (myositis type) (Answer)**
C. Orbital metastatic tumor
D. Cavernous hemangioma

Explanation: The extraocular muscle of myositis inflammatory pseudotumor was thickened, but it was mainly unilateral and single involvement, and the tendon and the muscle of the abdomen were involved at the same time. TED is more common in multiple extraocular muscles, with thickened muscle bellies and normal tendons.

(Moderate) Q11. For mild, inactive TED, what is the most important foundational treatment recommendation? [Single Choice]

A. **Strict smoking cessation (Answer)**
B. Immediate low-dose oral corticosteroids
C. Radiotherapy
D. Early surgical intervention

Explanation: Mild and inactive thyroid ophthalmopathy is mainly managed by observation and follow-up, control of risk factors, symptomatic and supportive treatment, and, if necessary, surgery to improve appearance.

(Difficult) Q12. For severe, sight-threatening active TED (e.g., DON), what is the most rapidly effective initial treatment? [Single Choice]

A. Orbital decompression surgery
B. Orbital radiotherapy
C. **Intravenous high-dose glucocorticoid pulse therapy (Answer)**
D. Immunosuppressants

Explanation: The first-line treatment of DON is glucocorticoid pulse therapy. Emergency treatment should be performed for patients with DON that is ineffective or partially responsive to treatment, and surgical decompression should be performed to reduce orbital pressure and relieve optic nerve pressure.

(Easy) Q13. What is an absolute contraindication for intravenous glucocorticoid pulse therapy? [Single Choice]

A. **Active viral hepatitis (Answer)**
B. Mild hyperglycemia
C. Mild hypertension
D. Osteoporosis

Explanation: Absolute contraindications of glucocorticoids include: allergic to glucocorticoids (GC); uncontrolled severe infection (such as active abscess, fungal infection) and no effective anti-infective treatment; a history of severe mental illness (GCs may induce or aggravate mental symptoms); active gastrointestinal bleeding (GCs can increase the risk of gastrointestinal mucosal injury); corneal ulcer (may aggravate ulcer progression).

(Easy) Q14. Regarding the thyroid function status of patients with TED, which statement is INCORRECT? [Single Choice]

A. Often associated with hyperthyroidism.

B. **TED does not occur in euthyroid patients. (Answer)**

C. A minority of patients are hypothyroid.

D. A minority of patients are euthyroid.

Explanation: Some patients with TED have normal thyroid function.

**Post-study author clarification**: Euthyroidism does not exclude TED. However, particularly when thyroid serology is also normal or the orbital presentation is atypical, the diagnosis requires careful clinical evaluation, appropriate orbital imaging, and exclusion of alternative causes.

(Moderate) Q15. Regarding the treatment sequencing principle for TED, which is correct? [Single Choice]

A. Strabismus surgery should be performed before orbital decompression surgery.

B. Eyelid surgery should be performed before orbital decompression and strabismus surgery.

C. **Disease activity should be controlled first, followed by management of structural sequelae in the stable phase. (Answer)**

D. All surgeries can be performed in a single session.

Explanation: The core of TED treatment: firstly, internal medicine, then surgery; the order principle of first decompression, then strabismus, and then eyelid.

(Easy) Q16. A 28-year-old male patient with newly diagnosed Graves’ disease and hyperthyroidism. He has no significant ocular complaints. Examination reveals mild widening of the palpebral fissures bilaterally, proptosis of 17mm OD and 18mm OS, and a CAS score of 1. What is the best management? [Single Choice]

A. **Actively control hyperthyroidism, smoking cessation, supportive eye care, and regular follow-up. (Answer)**
B. Intravenous glucocorticoid pulse therapy
C. Oral glucocorticoids
D. Orbital decompression surgery

Explanation: Mild and inactive TED is mainly managed by observation and follow-up, risk factor control, symptomatic and supportive treatment, and, if necessary, surgery to improve appearance.

(Moderate) Q17. A 60-year-old female patient with severe active TED and DON has poorly controlled diabetes mellitus. After one week of IV glucocorticoid pulse therapy, the response is poor, and glycemic control worsens. What is the most reasonable next treatment option? [Single Choice]

A. Increase the glucocorticoid dose.
B. Add oral hypoglycemic agents.
C. **Proceed with urgent orbital decompression surgery as soon as possible (Answer)**
D. Observation.

Explanation: The first-line treatment for DON is glucocorticoid pulse therapy. DON with ineffective treatment or contraindications to hormone therapy should be treated urgently, and surgical decompression should be performed to reduce orbital pressure and relieve optic nerve pressure.

(Moderate) Q18. A 45-year-old male patient is in the inactive phase of TED. His primary complaint is limited upgaze and diplopia due to fibrosis of the inferior rectus muscle. What is the best treatment? [Single Choice]

A. Intravenous glucocorticoid pulse therapy.
B. Orbital radiotherapy.
C. Orbital fat decompression.
D. **Inferior rectus recession surgery (Answer).**

Explanation: In the inactive phase of TED, restrictive strabismus may be considered for strabismus surgery. The purpose of the surgery is to solve strabismus and head position, and to eliminate fronto-frontal diplopia. It is challenging to eliminate diplopia in all directions. Surgical method: the first choice of the affected muscle is back surgery, not for antagonistic muscle shortening surgery.

(Easy) Q19. A 50-year-old female patient underwent bilateral balanced orbital decompression for TED 6 months ago. She is now euthyroid and in the inactive disease phase. She still has eyelid retraction, which is cosmetically concerning (photo provided). Corneal exposure symptoms were relieved by the decompression. What should be the next step? [Single Choice]

A. Repeat decompression surgery.

B. Strabismus correction surgery.

C. **Eyelid retraction repair surgery. (Answer)**

D. Botulinum toxin injection.

Explanation: Comprehensive application of the principle of treatment order, after the inflammation is static and the decompression is completed, the appearance of the eyelid is treated.

(Difficult) Q20. According to the international EUGOGO guidelines, what is the classic regimen for intravenous glucocorticoid pulse therapy in moderate-to-severe active TED? [Single Choice]

A. **Cumulative dose 4.5g weekly regimen (e.g., 0.5g weekly x 6 weeks, then 0.25g weekly x 6 weeks). (Answer)**

B. 500mg methylprednisolone once weekly for 6 weeks.

C. 500mg methylprednisolone once weekly for 6 weeks, followed by a tapering course of oral corticosteroids.

D. 1000mg methylprednisolone daily for 3 consecutive days.

Explanation: For moderate and active TED, the first-line treatment regimen is intravenous glucocorticoid shock therapy, and the classic regimen is weekly therapy with a cumulative dose of 4.5g (e.g., 0.5 g weekly for 6 weeks + 0.25 g weekly for 6 weeks).

**Post-study author clarification**: The 4.5 g intravenous methylprednisolone schedule stated in this item (0.5 g weekly for 6 weeks followed by 0.25 g weekly for 6 weeks) is the standard 12-week intravenous methylprednisolone regimen described in the 2021 EUGOGO guideline. However, the original explanation did not sufficiently distinguish this glucocorticoid dosing regimen from the complete first-line treatment recommendation. For most patients with moderate-to-severe, active TED, the 2021 EUGOGO guideline recommends intravenous methylprednisolone in combination with mycophenolate sodium (or mofetil) as first-line treatment. Higher-dose intravenous methylprednisolone monotherapy may be considered in selected more severe cases. The original question and answer have been retained because they reproduce the assessment actually administered to participants.

**Supplementary Table S4.** Online experiment assessing knowledge of Thyroid Eye Disease (TED) 1 week after watching the teaching video.

Dear participants,

Thank you for participating in the experiment, which aims to assess trainees’ and junior residents’ knowledge of thyroid eye disease (TED) after watching the teaching video within 2 weeks. The experiment is anonymous. All data collected will be used exclusively for this experiment and not for any other purposes.

Please complete the following questions **based on your current understanding, without recourse to online resources**. Your honest and complete responses are crucial to the validity of this research.

We greatly appreciate your time and invaluable assistance in this online experiment despite your busy schedule. Thank you for your cooperation!

The Research Team

Part 1. Personal information collection

Q1. Your Department and Position [Single-choice Question]

A. Ophthalmology Resident (Standardized Training)
B. General Resident (Standardized Training)

Q2. Your Gender [Single-choice Question]

C. Male
D. Female

Q3. Your Age: ___ years old. [Fill-in-the-blank]

Q4. Your initials: ___. (e.g., San Zhang: sz. It’s for distinguishing respondents only and will not disclose any personal information. Thank you for your cooperation!) [Fill-in-the-blank]

Part 2. Clinical questions about TED (including answers and explanations).

(Easy) Q1. What is the most common symptom of TED?

A. **Foreign body sensation, photophobia, and dryness(Answer)**

B. Eye pain

C. Decreased visual acuity

D. Diplopia

Explanation: The most common and early symptoms of TED are irritation symptoms caused by ocular surface exposure and inflammation, such as foreign body sensation, photophobia, and dryness in the eye. Diplopia and decreased vision are serious complications.

(Moderate) Q2. What is the primary cellular target of the autoimmune attack in the orbit in TED?

A. Retinal cells

B. Optic nerve glial cells

C. **Fibroblasts (Answer)**

D. Vascular endothelial cells

Explanation: The pathogenesis of TED : TSHR stimulation of TRAb can activate cAMP production and pAKT level in orbital fibroblasts, and further promote the production of adipogenic factors and hyaluronic acid in orbital fibroblasts. Hyaluronic acid swelling can lead to edema of extraocular muscles and orbital adipose connective tissue, increase the volume of orbital adipose tissue, and eventually lead to increased intraocular pressure and exophthalmos.

(Easy) Q3. Which of the following is an indicator of disease severity rather than activity in TED?

A. **Proptosis (Answer)**

B. Conjunctival hyperemia

C. Eyelid edema

D. Pain with eye movement

Explanation: Conjunctival congestion, eyelid edema, and pain during eye movement are activity indicators (CAS score items), exophthalmos, diplopia, and visual acuity are severity indicators, and attention should be paid to identification.

(Moderate) Q4. What is the most controllable environmental factor with a definitive impact on the development and severity of TED?

A. High-iodine diet

B. **Smoking (Answer)**

C. Psychological stress

D. UV exposure

Explanation: Studies have confirmed that smoking has a key negative effect on the pathogenesis and treatment response of TED, and is an important part of patient education.

(Moderate) Q5. TED often causes extraocular muscle enlargement. Which of the following statements is INCORRECT?

A. It often involves multiple extraocular muscles

B. The most frequent order of muscle involvement is inferior rectus, medial rectus, superior rectus, and lateral rectus

C. Ocular motility disturbance in TED is restrictive

D. **Isolated enlargement of only the lateral rectus muscle is commonly observed (Answer)**

Explanation: TED is more common in multiple extraocular muscles. CT showed thickening of the muscle belly and normal tendon.

(Moderate) Q6. Which of the following is NOT a characteristic feature of Dysthyroid Optic Neuropathy (DON)?

A. Decreased visual acuity

B. Visual field constriction or pathological scotomas

C. Optic disc edema or pallor

D. **Retinal detachment (Answer)**

Explanation: The characteristics of DON include decreased visual acuity, increased intraocular pressure, decreased color vision, decreased contrast sensitivity, positive relative afferent pupillary disorder (RAPD), optic disc changes, visual field defects, abnormal visual evoked potential ( VEP ), but not retinal detachment.

(Easy) Q7. What is the most important initial laboratory investigation for diagnosing TED?

A. Complete blood count

B. Liver and kidney function tests

C. **Thyroid function tests (including TSH, FT3, FT4) and TRAb (Answer)**

D. Erythrocyte sedimentation rate (ESR)

Explanation: The pathogenesis of TED is closely related to TRAb and abnormal thyroid function. Therefore, thyroid function tests ( including TSH, FT3, FT4 ) and thyroid antibody tests such as TRAb must be performed.

(Easy) Q8. A patient presents with a fixed globe in the horizontal plane, unable to perform adduction or abduction. Which muscle(s) are most likely involved?

A. Inferior and superior rectus muscles

B. **Medial and lateral rectus muscles (Answer)**

C. Superior and inferior oblique muscles

D. Levator palpebrae superioris muscle

Explanation: During the development of TED, any extraocular muscle may be involved, the extraocular muscles that control the inward and outward rotation of the eyeball are the medial rectus and the lateral rectus, respectively.

(Difficult) Q9. An elderly patient presents with progressive, painless unilateral proptosis accompanied by gradual vision loss. The most suspected diagnosis is:

A. Thyroid Eye Disease

B. Orbital inflammatory pseudotumor

C. **Orbital tumor (e.g., lymphoma, metastasis) (Answer)**

D. Orbital cellulitis

Explanation: Orbital tumors can occur unilaterally or bilaterally, and have nothing to do with thyroid function. Symptoms depend on the type, size and location of the tumor, often manifested as painless exophthalmos, movement disorders, decreased vision, and eye pain. Some tumors may be accompanied by facial numbness, headache and other symptoms. TED generally occurs in both eyes.

(Difficult) Q10. Compared to TED, which feature is NOT typical of orbital myositis (a subtype of inflammatory pseudotumor)?

A. More acute onset with severe pain

B. **Poor response to corticosteroid therapy (Answer)**

C. Often unilateral

D. Simultaneous thickening of both the tendon and belly of extraocular muscles

Explanation: Myositis-type inflammatory pseudotumor is mostly unilateral, with more acute onset, more obvious pain, effective glucocorticoids, but easy to relapse ; the extraocular muscles of the eye were often thickened, but the unilateral and single involvement was the main one, and the tendon and the muscle belly were involved at the same time. TED is a chronic disease with multiple eyes. Glucocorticoid is effective for active TED, with multiple extraocular muscles involved, thickened muscle belly, and normal tendon.

(Moderate) Q11. For patients with TED of all severity levels, what is the foundational treatment that should be initiated first?

A. Intravenous glucocorticoid pulse therapy

B. **Effective topical lubrication (e.g., artificial tears, gels) (Answer)**

C. Radiotherapy

D. Immunosuppressants

Explanation: Supportive therapy is the basic treatment for the management of all patients with TED.

(Easy) Q12. For moderate-to-severe active Thyroid Eye Disease, if a patient has contraindications to intravenous glucocorticoid therapy, what is a considered alternative?

A. **Orbital radiotherapy and/or immunosuppressants (Answer)**

B. Observation

C. Oral non-steroidal anti-inflammatory drugs

D. Immediate surgery

Explanation: Second-line treatment for patients with active TED, including immunosuppressive agents ( such as cyclosporine, mycophenolate mofetil ), biological agents ( such as tentuzumab, rituximab ), orbital radiotherapy, etc.

(Difficult) Q13. If a patient is completely euthyroid, what is the most valuable sign required for diagnosing TED?

A. Eyelid swelling

B. Proptosis

**C. Eyelid retraction (Answer)**

D. Extraocular muscle enlargement

Explanation: Eyelid retraction is the most diagnostic sign of thyroid eye disease.

**Post-study author clarification**: This original explanation was an oversimplification. Euthyroidism does not exclude TED, but no single clinical sign is independently required or sufficient to establish the diagnosis of TED in a euthyroid patient. Diagnosis should integrate the characteristic clinical presentation, thyroid/autoimmune evaluation, and, when appropriate, orbital imaging while excluding alternative orbital disorders. The 2021 EUGOGO guideline specifically identifies orbital MRI as indicated in patients with suspected GO presenting with euthyroidism and normal thyroid serology. The original item is retained to preserve the assessment exactly as administered to participants.

(Easy) Q14. For patients with active moderate-to-severe TED who are unresponsive or resistant to glucocorticoid therapy, which biologic agent(s) can be considered?

A. Only Teprotumumab can be chosen

B. Only Tocilizumab can be chosen

C. Biologics are not recommended

D. **Either Teprotumumab or Tocilizumab can be considered (Answer)**

Explanation: The consensus states that for patients who are resistant or resistant to glucocorticoids, biological agents such as tetuzumab or tocilizumab can be selected for treatment.

(Easy) Q15. Which statement regarding the surgical treatment of TED is correct?

A. **The typical surgical sequence is: Orbital decompression > Strabismus surgery > Eyelid surgery (Answer)**

B. Decompression surgery can be performed immediately upon diagnosis

C. Eyelid surgery should take precedence over orbital decompression

D. All surgeries should be performed in a single session during the active phase

Explanation: The core of TED treatment: internal medicine first, then surgery, and the order principle of first decompression, then strabismus, and then eyelid.

(Difficult) Q16. A 35-year-old female with a 1-year history of hyperthyroidism. Over the past 2 months, she has experienced worsening bilateral foreign body sensation and tearing. Examination reveals mild bilateral eyelid retraction, proptosis of 19mm OD and 20mm OS, and mild conjunctival hyperemia. CAS = 2. Visual acuity and visual fields are normal. What is the best management strategy?

A. High-dose intravenous glucocorticoids

B. Oral prednisone 30mg/day

C. **Strict smoking cessation, artificial tears, regular follow-up (Answer)**

D. Orbital decompression surgery

Explanation: Mild and inactive TED is mainly based on observation and follow-up, control of risk factors and symptomatic and supportive treatment, and surgery is required to improve the appearance if necessary.

(Moderate) Q17. A 40-year-old male with active TED (CAS=5) and mild compressive optic neuropathy. After completing one course of intravenous glucocorticoid pulse therapy, inflammatory signs improved significantly, but the optic neuropathy did not. Orbital CT is shown below. What is the most reasonable next step?

A. Immediately start a second course of glucocorticoid pulse therapy

B. Switch to oral immunosuppressants

C. Proceed with orbital radiotherapy

D. **Perform orbital decompression surgery as soon as possible (Answer)**

Explanation: The first-line treatment for DON is hormone shock. DON with ineffective treatment or contraindications to hormone therapy should be treated urgently, and surgical decompression should be performed to reduce orbital pressure and relieve optic nerve pressure.

(Moderate) Q18. A 35-year-old female in the inactive phase of TED. She has previously undergone orbital decompression and strabismus surgery. Her current stable issue is mild bilateral upper eyelid retraction causing cosmetic concern. What is the preferred treatment?

A. Botulinum toxin injection

B. **Müller's muscle excision (Answer)**

C. Glucocorticoid pulse therapy

D. Repeat orbital decompression

Explanation: The choice of surgical methods for blepharoplasty: Müller myectomy (mild upper eyelid retraction, 1-2mm), levator lengthening (moderate upper eyelid retraction, 3-4mm),levator myotomy (moderate to severe upper eyelid retraction), combined surgery (moderate to severe upper eyelid retraction), implant filling surgery (severe upper eyelid retraction,≥5mm).

(Difficult) Q19. A 52-year-old male receiving intravenous glucocorticoid pulse therapy for active TED. After the second infusion, he develops sudden increased intraocular pressure, severe eye pain accompanied by ipsilateral headache, nausea, and vomiting. What is the most likely diagnosis?

A. **Acute angle-closure glaucoma (Answer)**

B. Acute exacerbation of Thyroid Eye Disease

C. Optic neuritis

D. Corneal infection

Explanation: Hormone therapy may induce acute angle-closure glaucoma, which is in line with the signs of sudden increase in intraocular pressure. The symptoms of TED itself generally do not cause severe eye pain, headache, nausea, vomiting and so on.

(Difficult) Q20. According to the EUGOGO guidelines, what is the recommended treatment strategy for mild TED?

A. Oral glucocorticoids

B. Local glucocorticoid injections

C. Prophylactic orbital radiotherapy

D. **Non-immunosuppressive supportive and symptomatic treatment (Answer)**

Explanation: Mild and inactive thyroid eye disease is mainly based on observation and follow-up, control of risk factors and symptomatic and supportive treatment, and surgery is required to improve the appearance if necessary.

**Supplementary Table S5.** Informed Consent Form

**Study Title:** Application of AI-Generated Teaching Videos in Thyroid Eye Disease (TED) Education
**Principal Investigator:** Shu Liu, Shanghai Changzheng Hospital
**Contact Email:** liu970105@163.com

**1. Purpose of the Study**

We cordially invite you to participate in a teaching study. Before you decide whether to take part, please read the following information carefully and feel free to ask the researcher any questions.

This study aims to compare the educational effectiveness of artificial intelligence (AI)-generated teaching videos versus teacher‑made videos in thyroid eye disease (TED) education.

**2. Voluntary Participation**

Your participation in this study is entirely voluntary. You may decide not to participate or withdraw at any time without providing any reason, and this will not negatively affect your training, assessment, or professional development.

**3. Study Procedures**

If you agree to participate, you will be asked to complete a series of short online video learning activities and tests, which will be administered at three different time points. Before you begin, we will clearly inform you of the total time required, and you will have the opportunity to ask questions about the specific arrangements.

**4. Potential Risks and Benefits**

This study involves minimal risk, similar to routine online learning tasks. Some participants may experience mild eye fatigue or boredom. You may take a break whenever needed. If you feel significant discomfort, you may withdraw from the study at any time.

This study may not provide direct benefit to you personally. However, your participation will help researchers better understand how AI‑generated educational tools can support medical training, thereby benefiting future learners and educators.

**5. Confidentiality**

All collected data will be anonymized and stored securely. Your personal information will not be shared or published. The study results will be reported only in aggregate form, and no individual participant will be identifiable.

**6. Contact Information**

If you have further questions about the study, please contact the principal investigator, Shu Liu, at liu970105@163.com.

**7. Statements of Consent**

**Participant’s Statement:**

I have carefully read the above information (or it has been read to me). The research team has fully explained the purpose, procedures, risks, and protections of this study to me. I understand the nature of this study. I voluntarily agree to participate and will truthfully complete all required study activities.

**Participant’s Signature:** _________________
**Date:** ___________ (DD/MM/YYYY)

**Investigator’s Statement:**

I have fully explained the content of this study to the participant and confirm that the participant understands the above information.

**Investigator’s Signature:** _________________
**Date:** ___________ (DD/MM/YYYY)

**Supplementary Table S6. The CVI of expert video content validity assessment scale.**

| Variable | Questions | I-CVI | | Average Scores | |
| --- | --- | --- | --- | --- | --- |
|  |  | AI-assisted | Teacher-made | AI-assisted | Teacher-made |
| Clinical Accuracy | 1.The information regarding TED epidemiology and pathogenesis is accurate  2.The diagnostic criteria and treatment approaches presented in the video are consistent with current clinical practice.  3.The description of clinical signs and their interpretation is clinically correct.  4. No factual medical errors or potentially misleading statements were identified in the video. | 1.00  1.00  1.00  0.83 | 1.00  1.00  1.00  1.00 | 4.50  4.50  4.83  4.00 | 5.00  4.67  4.50  4.17 |
| Visual Fidelity | 1. The anatomical structures are accurately depicted and clearly distinguishable.  2.The pathological changes are visually authentic and realistic.  3.The images or animations are anatomically accurate and free from distortion.  4.The visual representations appropriately support the verbal content and enhance understanding of the clinical concepts. | 0.83  0.83  0.67  1.00 | 0.83  0.83  1.00  1.00 | 4.17  3.83  4.33  4.33 | 4.17  4.17  4.33  4.50 |
| Pedagogical Appropriateness | 1.The video content is logically structured and follows a clear learning pathway.  2.The language, terminology, and explanations are appropriate for the residents.  3.The pacing, duration, and visual-verbal balance are suitable for effective learning  4.The video effectively highlights key concepts of TED. | 1.00  1.00  1.00  1.00 | 1.00  1.00  1.00  1.00 | 4.50  4.67  4.67  4.83 | 4.83  4.67  4.83  4.83 |

In the rating items, 1-Strongly Disagree, 2-Disagree, 3-Neither Agree nor Disagree, 4-Agree, 5-Strongly Agree.

**Supplementary Table S7. The CVI of expert assessment scale for the three test.**

| Questions | I-CVI | | | Average Scores | | |
| --- | --- | --- | --- | --- | --- | --- |
|  | Test 1 | Tset 2 | Test 3 | Test 1 | Test 2 | Test 3 |
| 1.The three tests collectively cover the core knowledge areas of thyroid eye disease (TED)  2.The proportions of easy, moderate, and difficult items are consistent across the three tests.  3.The overall difficulty levels of the three tests are comparable.  4. The items in all three tests are clearly worded and unambiguous, making them easy for trainees to understand.  5.The length of each test (20 items) and the time required to complete it are appropriate. | 1.00  0.83  1.00  1.00  1.00 | 1.00  1.00  1.00  1.00  1.00 | 0.83  1.00  1.00  1.00  1.00 | 4.50  4.17  4.83  4.67  4.50 | 4.67  4.33  4.50  4.67  4.67 | 4.17  4.33  4.67  4.50  4.50 |

In the rating items, 1-Strongly Disagree, 2-Disagree, 3-Neither Agree nor Disagree, 4-Agree, 5-Strongly Agree.

**Supplementary Table S8.** Item-level and mean difficulty and discrimination for the three test forms.

| **Item** | **Test 1** | | | **Test 2** | | | **Test 3** | | |
| --- | --- | --- | --- | --- | --- | --- | --- | --- | --- |
|  | Difficulty (p) | Discrimination (27%) | Point-Biserial | Difficulty (p) | Discrimination (27%) | Point-Biserial | Difficulty (p) | Discrimination (27%) | Point-Biserial |
| 1 | 0.43 | 0.75 | 0.49 | 0.87 | 0.42 | 0.50 | 0.74 | 0.42 | 0.24 |
| 2 | 0.67 | 0.83 | 0.58 | 0.93 | 0.25 | 0.40 | 0.74 | 0.75 | 0.60 |
| 3 | 0.89 | 0.25 | 0.29 | 0.61 | 0.17 | -0.11 | 0.83 | 0.50 | 0.52 |
| 4 | 0.70 | 0.42 | 0.28 | 0.80 | 0.50 | 0.40 | 0.65 | 0.83 | 0.68 |
| 5 | 0.35 | 0.67 | 0.47 | 1.00 | 0.00 | NA | 0.70 | 0.58 | 0.37 |
| 6 | 0.59 | 0.58 | 0.36 | 0.85 | 0.42 | 0.35 | 0.70 | 0.75 | 0.58 |
| 7 | 0.54 | 0.92 | 0.65 | 0.70 | 0.67 | 0.61 | 1.00 | 0.00 | NA |
| 8 | 0.85 | 0.33 | 0.17 | 0.67 | 0.75 | 0.63 | 0.76 | 0.17 | 0.09 |
| 9 | 0.65 | 0.33 | 0.30 | 0.74 | 0.42 | 0.28 | 0.54 | 0.42 | 0.23 |
| 10 | 0.74 | 0.58 | 0.38 | 0.65 | 0.83 | 0.63 | 0.46 | 0.25 | 0.10 |
| 11 | 0.98 | 0.00 | 0.04 | 0.74 | 0.67 | 0.54 | 0.74 | 0.67 | 0.62 |
| 12 | 0.93 | 0.25 | 0.28 | 0.72 | 0.50 | 0.34 | 0.85 | 0.42 | 0.30 |
| 13 | 0.30 | 0.67 | 0.54 | 0.89 | 0.08 | 0.05 | 0.57 | 0.58 | 0.36 |
| 14 | 0.54 | 0.67 | 0.38 | 0.91 | 0.17 | 0.18 | 0.83 | 0.33 | 0.35 |
| 15 | 0.48 | 1.00 | 0.70 | 0.85 | 0.25 | 0.10 | 0.83 | 0.50 | 0.53 |
| 16 | 0.98 | 0.08 | 0.08 | 0.87 | 0.33 | 0.31 | 0.65 | 0.75 | 0.45 |
| 17 | 0.67 | 0.67 | 0.52 | 0.80 | 0.25 | 0.24 | 0.65 | 0.42 | 0.26 |
| 18 | 0.96 | 0.17 | 0.24 | 0.74 | 0.42 | 0.12 | 0.72 | 0.50 | 0.43 |
| 19 | 0.54 | 0.50 | 0.28 | 0.85 | 0.42 | 0.41 | 0.54 | 0.92 | 0.60 |
| 20 | 0.57 | 0.08 | −0.10 | 0.61 | 0.58 | 0.41 | 0.57 | 0.67 | 0.40 |
| **Mean** | **0.67** | **0.48** | **0.35** | **0.79** | **0.40** | **0.34** | **0.70** | **0.52** | **0.41** |

Item 5 and 7 had a difficulty of 1.00 (all participants answered correctly), so point-biserial correlation could not be computed (NA). Mean point-biserial is based on the 19 items with available values.

**Supplementary Table S9. Between-group differences in test scores (AI-assisted minus Teacher-made).**

| **Test** | **Analysis** | **Mean difference (95% CI)** | **p value (Bonferroni-adjusted)** | **Cohen’s d** |
| --- | --- | --- | --- | --- |
| Test 1 | Primary (n=46) | -4.64 (-15.45 to 6.17) | 0.40 | -0.35 |
|  | ITT (n=52) | -5.59 (-17.04 to 5.85) | 0.33 | -0.23 |
|  | Sensitivity (n=50) | -5.60 (-17.57 to 6.37) | 0.35 | -0.14 |
| Test 2 | Primary (n=46) | -3.12 (-13.92 to 7.69) | 0.57 | -0.44 |
|  | ITT (n=52) | -2.56 (-14.00 to 8.89) | 0.66 | -0.20 |
|  | Sensitivity (n=50) | -3.00 (-14.97 to 8.97) | 0.62 | -0.08 |
| Test 3 | Primary (n=46) | -1.81 (-12.62 to 9.00) | 0.74 | -0.43 |
|  | ITT (n=52) | -1.02 (-12.60 to 10.56) | 0.86 | -0.23 |
|  | Sensitivity (n=50) | -1.20 (-13.17 to 10.77) | 0.84 | -0.09 |

Mean differences are AI-assisted minus Teacher-made. p values are Bonferroni-adjusted. No significant between-group differences were detected.

**Supplementary Table S10. Within-group changes in test scores (later time point minus earlier time point).**

| **Group** | **Analysis** | **Comparison** | **Mean difference (95% CI)** | **p value (Bonferroni-adjusted)** |
| --- | --- | --- | --- | --- |
| AI-assisted | Primary (n=46) | Test 2 minus Test 1 | 13.04 (3.51 to 22.57) | 0.004 |
|  |  | Test 3 minus Test 1 | 7.61 (-1.92 to 17.14) | 0.16 |
|  |  | Test 3 minus Test 2 | -5.43 (-14.97 to 4.10) | 0.50 |
|  | ITT (n=52) | Test 2 minus Test 1 | 13.42 (4.81 to 22.04) | <0.001 |
|  |  | Test 3 minus Test 1 | 8.92 (0.18 to 17.66) | 0.04 |
|  |  | Test 3 minus Test 2 | -4.50 (-13.24 to 4.23) | 0.64 |
|  | Sensitivity (n=50) | Test 2 minus Test 1 | 13.20 (4.32 to 22.08) | 0.001 |
|  |  | Test 3 minus Test 1 | 8.80 (-0.08 to 17.68) | 0.05 |
|  |  | Test 3 minus Test 2 | -4.40 (-13.28 to 4.48) | 0.69 |
| Teacher-made | Primary (n=46) | Test 2 minus Test 1 | 11.52 (1.99 to 21.05) | 0.01 |
|  |  | Test 3 minus Test 1 | 4.78 (-4.75 to 14.31) | 0.67 |
|  |  | Test 3 minus Test 2 | -6.74 (-16.27 to 2.79) | 0.26 |
|  | ITT (n=52) | Test 2 minus Test 1 | 10.38 (1.77 to 19.00) | 0.01 |
|  |  | Test 3 minus Test 1 | 4.34 (-4.39 to 13.08) | 0.69 |
|  |  | Test 3 minus Test 2 | -6.04 (-14.78 to 2.69) | 0.29 |
|  | Sensitivity (n=50) | Test 2 minus Test 1 | 10.60 (1.72 to 19.48) | 0.01 |
|  |  | Test 3 minus Test 1 | 4.40 (-4.48 to 13.28) | 0.69 |
|  |  | Test 3 minus Test 2 | -6.20 (-15.08 to 2.68) | 0.28 |

### Mean differences are later time point minus earlier time point. Positive values indicate higher scores at the later time point. p values are Bonferroni-adjusted within each group.

**Supplementary Table S11.** **Between-group differences in completion time (AI-assisted minus teacher-made, log scale)**

| **Test** | **Analysis** | **Log mean difference (95% CI)** | **P value (Bonferroni-adjusted)** | **GMR (95% CI)** |
| --- | --- | --- | --- | --- |
| Test 2 | Primary (n=46) | -0.06 (-0.36 to 0.24) | 0.70 | 0.94 (0.70 to 1.28) |
|  | ITT (n=52) | -0.04 (-0.37 to 0.29) | 0.81 | 0.96 (0.69 to 1.34) |
|  | Sensitivity (n=50) | -0.12 (-0.46 to 0.22) | 0.47 | 0.89 (0.63 to 1.24) |
| Test 3 | Primary (n=46) | -0.09 (-0.39 to 0.21) | 0.56 | 0.92 (0.68 to 1.24) |
|  | ITT (n=52) | -0.07 (-0.41 to 0.26) | 0.67 | 0.93 (0.66 to 1.30) |
|  | Sensitivity (n=50) | -0.12 (-0.46 to 0.21) | 0.47 | 0.88 (0.63 to 1.24) |

Comparisons are AI-assisted minus Teacher-made.. Negative log mean differences and GMR < 1 indicate shorter completion time in the AI-assisted group. p values are Bonferroni-adjusted.

**Supplementary Table S12. Within-group changes in completion time (Test 3 minus Test 2, log scale).**

| **Group** | **Analysis** | **Log mean difference (95% CI)** | **P value (Bonferroni-adjusted)** | **GMR (95% CI)** |
| --- | --- | --- | --- | --- |
| AI-assisted | Primary (n=46) | -0.22 (-0.47 to 0.04) | 0.09 | 0.80 (0.62 to 1.04) |
|  | ITT (n=52) | -0.28 (-0.54 to -0.02) | 0.03 | 0.76 (0.59 to 1.00) |
|  | Sensitivity (n=50) | -0.25 (-0.51 to 0.00) | 0.05 | 0.78 (0.60 to 1.00) |
| Teacher-made | Primary (n=46) | -0.19 (-0.44 to 0.07) | 0.15 | 0.83 (0.64 to 1.07) |
|  | ITT (n=52) | -0.24 (-0.49 to 0.01) | 0.06 | 0.79 (0.61 to 1.01) |
|  | Sensitivity (n=50) | -0.25 (-0.50 to 0.00) | 0.05 | 0.78 (0.61 to 1.00) |

Comparisons are Test 3 minus Test 2. Negative log mean differences and GMR < 1 indicate shorter completion time at 1 week. p values are Bonferroni-adjusted.

**Supplementary Table S13.** Exploratory subgroup analysis of test scores by residency type.

| **Group** | **subgroup** | **Test 1** | **Test 2** | **Scores improvement [Test 2 minus Test 1]** | **Test 3** | **Scores improvement [Test 3 minus Test 1]** |
| --- | --- | --- | --- | --- | --- | --- |
| AI-assisted | Ophthalmology | 73.33±18.03 | 86.67±12.50 | 13.33±15.61 | 86.11±12.19 | 12.78±18.73 |
|  | General | 60.71±22.00 | 73.57±19.56 | 12.86±24.86 | 65.00±17.65 | 4.29±26.66 |
| Teacher-made | Ophthalmology | 75.00±12.50 | 90.00±7.07 | 15.00±11.46 | 88.33±10.31 | 13.33±13.23 |
|  | General | 63.57±20.14 | 72.86±16.84 | 9.29±17.08 | 62.86±18.47 | -0.71±21.83 |

Data are shown as mean±SD. Test 2 minus Test 1 = 5 min minus baseline; Test 3 minus Test 1 = 1 week minus baseline. Negative values indicate shorter completion time at follow-up.

**Supplementary Table S14.** Exploratory subgroup analysis of completion time by residency type.

| **Group** | **Subgroup** | **Test 1** | **Test 2** | **Time reduction [Test 2 minus Test 1]** | **Test 3** | **Time reduction**  **[Test 3 minus Test 1]** |
| --- | --- | --- | --- | --- | --- | --- |
| AI-assisted | Ophthalmology | 498.0 (289.2 to 922.0 | 256.0 (221.2 to 363.8) | -295.5 (-696.5 to -4.2) | 277.5 (198.0 to 397.0) | -318.0 (-691.5 to 7.5) |
|  | General | 700.0 (281.0 to 1012.0) | 401.0 (364.0 to 555.0) | -299.0 (-822.0 to 89.0) | 249.0 (195.0 to 323.0) | -343.0 (-801.0 to -86.0) |
| Teacher-made | Ophthalmology | 471.0 (304.0 to 562.0) | 420.0 (300.0 to 540.0) | -61.0 (-148.0 to 150.0) | 403.0 (200.0 to 625.0) | -37.0 (-163.0 to 55.0) |
|  | General | 467.0 (360.2 to 590.8) | 285.5 (259.5 to 355.0) | -125.0 (-303.0 to -33.2) | 206.0 (173.2 to 250.0) | -209.5 (-354.0 to -138.0) |

Data are median with IQRin seconds. Test 2 minus Test 1 = 5 min minus baseline; Test 3 minus Test 1 = 1 week minus baseline. Negative values indicate shorter completion time at follow-up.

**Supplementary Table S15. Themes from open-ended responses.**

| **Theme** | **AI-assisted (n = 13)** | **Teacher-made (n = 14)** | **Total (n = 27)** |
| --- | --- | --- | --- |
| Pacing | 6 (46.2%) | 7 (50.0%) | 13 (48.1%) |
| Content comprehensiveness | 5 (38.5%) | 4 (28.6%) | 9 (33.3%) |
| Visual quality | 3 (23.1%) | 4 (28.6%) | 7 (25.9%) |
| Suggestion: better pacing | 3 (23.1%) | 2 (14.3%) | 5 (18.5%) |
| Suggestion: more visual aids | 1 (7.7%) | 2 (14.3%) | 3 (11.1%) |
| Suggestion: more clinical cases | 1(7.7%) | 1(7.1%) | 2(7.4%) |

**Supplementary Figure S1.** Comparison of three‑round test accuracy for DON between subgroups. (A) Accuracy for residents who studied the AI-generated video. (B) Accuracy for residents who studied the teacher-made video. Significant improvements were noted in both groups following training. (ns = not significant).
